# Mechanical phenotyping reveals unique biomechanical responses in retinoic acid-resistant acute promyelocytic leukemia

**DOI:** 10.1016/j.isci.2022.103772

**Published:** 2022-01-15

**Authors:** Brian Li, Annie Maslan, Sean E. Kitayama, Corinne Pierce, Aaron M. Streets, Lydia L. Sohn

**Affiliations:** 1 UC Berkeley – UCSF Graduate Program in Bioengineering, Berkeley and San Francisco, CA 94709, USA; 2 Department of Molecular and Cellular Biology, University of California, Berkeley, Berkeley 94720, USA; 3 Center for Computational Biology, University of California, Berkeley, CA 94709, USA; 4 Chan-Zuckerberg Biohub, San Francisco, CA 94158, USA; 5 Department of Mechanical Engineering, University of California, Berkeley, CA 94720, USA

**Keywords:** Cellular physiology, Molecular biology, Cell biology, Biomechanics

## Abstract

All-trans retinoic acid (ATRA) is an essential therapy in the treatment of acute promyelocytic leukemia (APL), but nearly 20% of patients with APL are resistant to ATRA. As there are no biomarkers for ATRA resistance that yet exist, we investigated whether cell mechanics could be associated with this pathological phenotype. Using mechano-node-pore sensing, a single-cell mechanical phenotyping platform, and patient-derived APL cell lines, we discovered that ATRA-resistant APL cells are less mechanically pliable. By investigating how different subcellular components of APL cells contribute to whole-cell mechanical phenotype, we determined that nuclear mechanics strongly influence an APL cell’s mechanical response. Moreover, decondensing chromatin with trichostatin A is especially effective in softening ATRA-resistant APL cells. RNA-seq allowed us to compare the transcriptomic differences between ATRA-resistant and ATRA-responsive APL cells and highlighted gene expression changes that could be associated with mechanical changes. Overall, we have demonstrated the potential of “physical” biomarkers in identifying APL resistance.

## Highlights

• Mechanical phenotyping of acute promyelocytic leukemia (APL) cells
• All-trans retinoic acid (ATRA) resistance in APL is associated with cell rigidity
• RNA-seq of APL cells reveals genes that may potentially contribute to cell mechanics

## Graphical abstract

**Figure:**
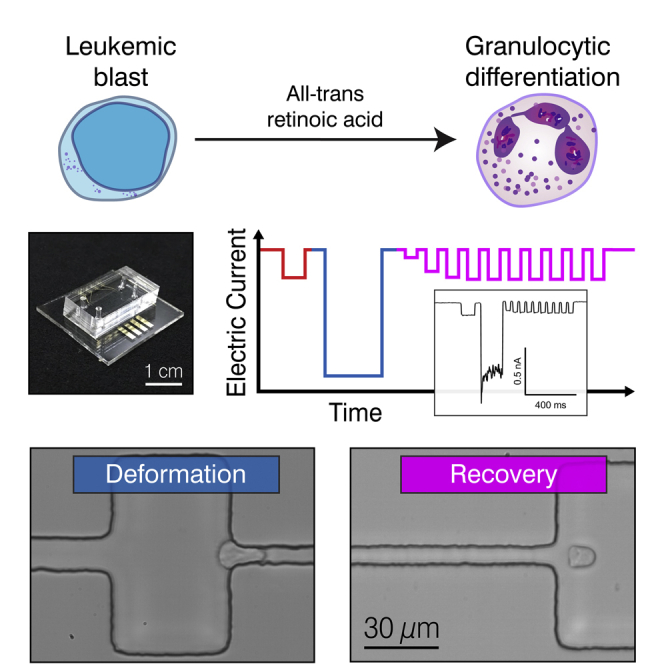

## Introduction

Physical and mechanical properties such as mass growth rate and deformability are increasingly being explored as possible biomarkers of cancer cells (Byun et al., 2013; Gossett et al., 2012; Kim et al., 2018; Kimmerling et al., 2018; Toepfner et al., 2018; Tse et al., 2013). Although cells from solid tumors have been the primary focus of these studies, those from liquid tumors have also been investigated, albeit to a lesser extent. Atomic force microscopy and optical stretcher studies have shown that deformability and mechanical compliance are important in the pathological dissemination of leukemic cells throughout the circulatory system (Lam et al., 2007; Lautenschläger et al., 2009; Pals et al., 2007; Rosenbluth et al., 2006; Stein et al., 2009). More recently, studies involving suspended microchannel resonators have correlated physical properties (e.g., cell mass and growth rate) of leukemias and lymphomas to important pathological processes such as drug sensitivity or proliferation (Mu et al., 2020; Stevens et al., 2016) and have determined the biophysical factors that influence cell mechanical properties, such actin remodeling and cell cycle phase (Kang et al., 2019). Despite these investigations, the relevance of the physical and mechanical attributes of liquid tumor cells to sensitivity to therapy has yet to be examined (Lam et al., 2007; Stevens et al., 2016).

Here, we assess the cell mechanics of acute promyelocytic leukemia (APL), an acute myeloid leukemia (AML) subtype characterized by a fusion gene between PML (promyelocytic leukemia protein) and RARA (retinoic acid receptor alpha) (Rowley et al., 1977). In APL, the PML-RARA fusion gene occurs in granulocytic precursor cells known as promyelocytes, halting their differentiation and causing rapid proliferation of the immature blasts. Because of the PML-RARA fusion gene, the standard of care for APL patients is to administer all-trans retinoic acid (ATRA), which induces differentiation of the promyelocytes and, in turn, resolves the acute phase of the disease (Douer et al., 2013; Mendez et al., 2018; Puccetti and Ruthardt, 2004; Tallman et al., 1997). This acute phase can rapidly progress to mortality within a week if left untreated. Early deaths due to disseminated intravascular coagulopathy are of especially great risk in *ATRA-resistant* cases, which comprise nearly 20% of all APL cases (Douer et al., 1996; Park et al., 2011; Ribeiro and Rego, 2006; Stein et al., 2009). Although APL has been well characterized biochemically using cDNA microarrays and RT-PCR (Gallagher, 2002; Lee et al., 2002; Noguera et al., 2019; Yang et al., 2003), there has only been limited characterization of APL cell mechanics. Specifically, Lautenschläger et al. showed that the APL cell line, NB4, becomes more deformable with ATRA treatment as it differentiates (Lautenschläger et al., 2009). However, beyond this work, there have been no investigations of the mechanical responses of ATRA-resistant APL to our knowledge.

We used mechano-node-pore sensing (mechano-NPS) (Kim et al., 2018), a microfluidic platform that utilizes a narrow channel to deform cells and measure their mechanical properties, to investigate ATRA-sensitive and ATRA-resistant APL cells. Through our mechano-NPS measurements, we determined that ATRA-resistant APL cells are significantly stiffer than ATRA-sensitive APL cells and are influenced by the organization of structural subcellular components such as the nucleus and cytoskeletal proteins. Using chemical cell cycle arrest and perturbation of chromatin accessibility, we evaluated how both major and minor changes in nuclear structure, respectively, affect APL mechanical phenotypes. We discovered that only ATRA-resistant APL cells are measurably softened after pharmacological chromatin decondensation. By performing RNA-seq analysis of ATRA-resistant and ATRA-sensitive cells, we have obtained a whole-transcriptome perspective of how gene expression changes in response to ATRA. This work, in particular, serves as a resource for further studies of ATRA resistance in APL. Overall, our studies show that ATRA resistance in APL corresponds to a state of cell rigidity that can be measured with mechano-NPS.

## Results

### Single-cell mechanical phenotyping of APL cells

**Figure 1:**
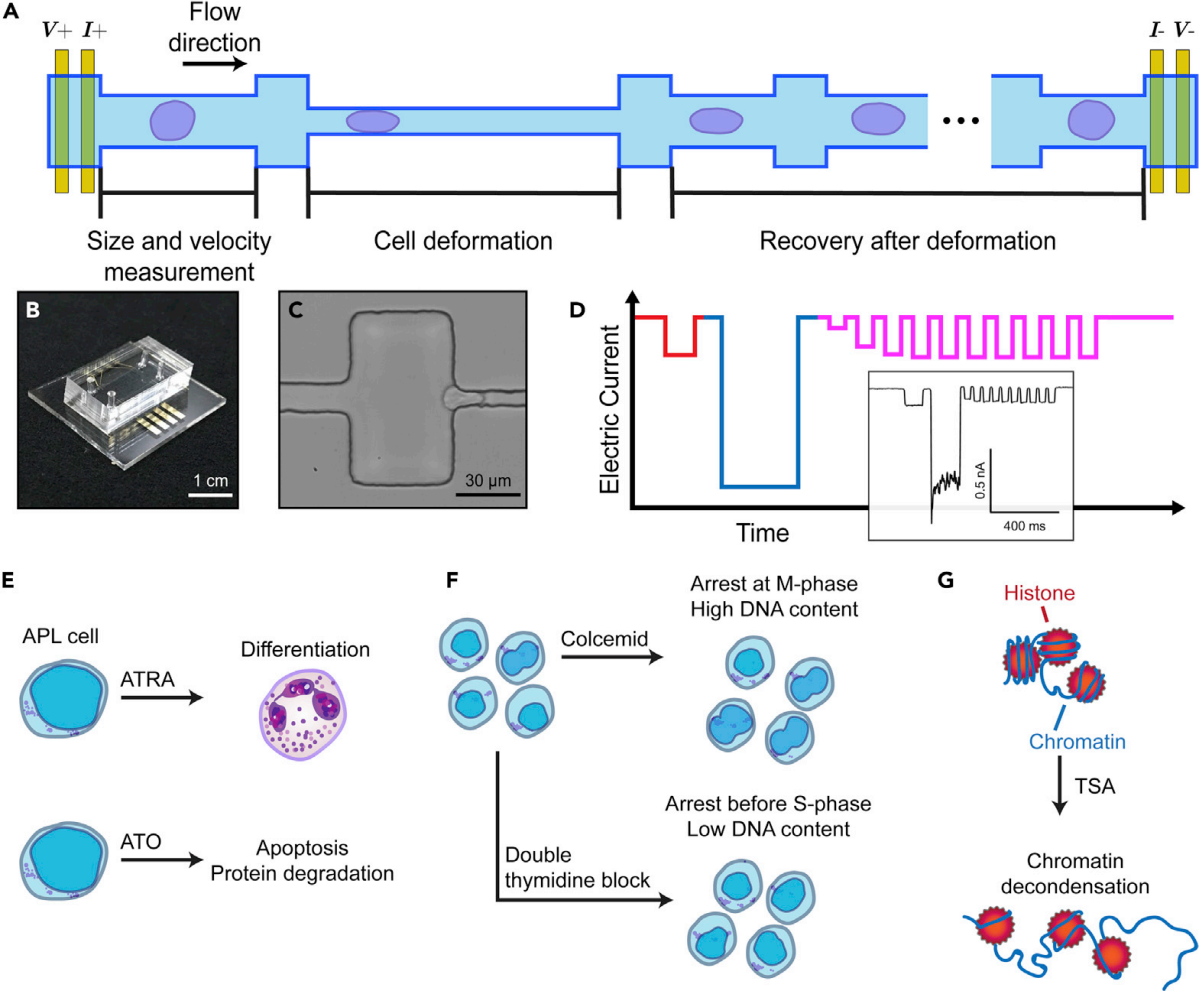
Overview of mechano-node-pore sensing (mechano-NPS) platform (A) Top view schematic of mechano-NPS microchannel. A four-terminal measurement is used to measure the current across the microchannel. Cell size (*d*_0_) and velocity (*u*_*flow*_) are measured prior to cell deformation. During deformation, the transit time of a cell is dependent on the cell’s elastic modulus. After it leaves the contraction channel, the cell recovers from an ellipsoid shape to a sphere. (B**)** Photograph of a mechano-NPS platform, with a polydimethylsiloxane (PDMS) mold embedded with two mechano-NPS channels and bonded to a glass substrate with prefabricated metal electrodes. (C**)** High-speed image of an NB4 cell deforming in transit through the contraction channel. (D**)** Expected current pulse caused by a cell transiting the entire mechano-NPS channel. The current pulse consists of subpulses that reflect the three main regions of the microchannel. (1) In the initial cell sizing segment (red), the free diameter and transit time of the cell are measured by the subpulse amplitude and duration, respectively. (2) In the contraction segment (blue), the transit time of the cell is measured by the subpulse duration, where more deformable cells transit this segment faster. (3) In the 10 cell recovery segments (magenta), cells recover from a deformed ellipsoid shape to a sphere, which is reflected in the gradual increase in subpulse amplitude over time. The inset shows an actual current pulse produced by an NB4 cell transiting a 6-mm-long mechano-NPS channel at an inlet pressure of 80 mbar. (E–G). Schematics of the major intracellular perturbations made in this study and their targets. These drugs were used to (E) test APL resistance to differentiation, (F) perturb DNA content via cell cycle arrest, and (G) decondense chromatin with a histone deacetylase inhibitor. ATRA, all-trans retinoic acid; ATO, arsenic trioxide; TSA, trichostatin A. See also Video S1 and Figure S1

Mechano-NPS (Kim et al., 2018) is an electronic method for mechanophenotyping cells that involves measuring the current across a microfluidic channel segmented by widened “nodes” (Figures 1A and 1B; see STAR Methods for fabrication and platform operation details). The width of one segment is narrower than a cell diameter (hereafter referred to as the “contraction” segment), and cells must squeeze through this segment in order to traverse the entire channel (Figure 1C). Wider “recovery” segments immediately following the contraction segment allow the cell to recover from deformation. When a cell transits the channel, a unique current pulse is measured. This current pulse is composed of subpulses that correspond to the cell traversing specific mechano-NPS segments (Figure 1D) (see STAR Methods). It is these subpulses from which physical and mechanical properties of the cell can be extracted. Specifically, the magnitude and duration of the subpulse prior to the cell entering the contraction segment reflect the cell size and transit time, respectively. The dramatically larger subpulse corresponds to the cell entering the contraction segment, and its duration provides information on the cell’s deformability (i.e., a more deformable cell will transit the contraction segment faster than one that is stiffer). Finally, the series of subpulses produced by the cell transiting the series of node-pores following the contraction segment tracks the cell’s recovery from deformation. As the cell relaxes back to its original size and shape, the magnitude of the subpulses return to that of the initial subpulse, i.e., the one caused by the cell initially entering the first node-pore segment of the device (Video S1).

Video S1. AP-1060 cell exiting the contraction segment of a mechano-NPS channel, related to Figure 1 Cells exit this segment deformed into a bullet shape due to a combination of shear stress and compressive stresses from the channel side walls. Mechano-NPS measures the relaxation from this bullet shape to a more spherical morphology to quantify a cell’s recovery from deformation. Contraction segment width is 7 μm, node (wide channel) width is 85 μm, and recovery segment width is 13 μm.

A cell’s mechanical response is dependent on the degree of applied strain, *ε*:

(Equation 1) $$\varepsilon =\left(d_{0}-d_{c}\right)/d_{0}$$

where *d*_0_ is the cell’s initial diameter and $d_{c}$ is the cell’s deformed diameter. For our studies, *ε* ≈ 0.35 in the contraction segment. To describe a cell’s deformability, we use the whole-cell deformability index (*wCDI*), a dimensionless parameter that relates a cell’s size and transit time through the contraction segment (Kim et al., 2018):

(Equation 2) $$wCDI=\frac{l_{c}}{u_{\text{flow}}\times h_{\text{channel}}}\times \frac{d_{\text{cell}}}{t_{\text{cont}}}$$

where *l*_*c*_ is the length of contraction segment, *u*_flow_ is the cell’s velocity in the pore before the contraction segment, *h*_channel_ is the height of the contraction segment, *d*_cell_ is the cell’s initial diameter, and *t*_cont_ is the transit time through the contraction segment. As Kim et al. established with micropipette aspiration studies, the *wCDI* is inversely related to the cell’s cortical tension (Kim et al., 2018), and cells that are softer will have a higher *wCDI* than those that are stiffer. Moreover, the *wCDI* negates cell-size effects: larger cells will naturally take longer to transit the contraction channel compared with smaller cells with similar stiffnesses. To ensure that the friction between channel walls and cell surfaces remained constant in our measurements, we flowed all cells in PBS supplemented with 2% fetal bovine serum (see STAR Methods).

As cells are intrinsically viscoelastic materials, the time-dependent response to changes in applied stress is a critical aspect of cell mechanical phenotype. To measure cell viscoelastic behavior, we use the recovery segments in our mechano-NPS channel to quantify how cells recover from deformation. For the experiments we report here, we designed a device with 10 recovery channel segments, which provided sufficient information to calculate the time constant of a cell’s recovery using linear least squares regression for a Kelvin-Voigt model (see STAR Methods). This recovery time ranged from 20 to 800 ms, with longer recovery times indicative of more viscous behavior (Figure S1).

### AP-1060 cells resist differentiation in ATRA and are more mechanically rigid

Of the only three cell lines established from patients with APL, we investigated two—NB4 and AP-160—which have varying degrees of sensitivity to ATRA and from which the majority of published APL studies have been performed (Abecassis et al., 2008; Lautenschläger et al., 2009; Long et al., 2010; Zhu et al., 2020). NB4 was derived from a patient who retained the characteristic sensitivity to ATRA differentiation, and AP-1060 was derived from an individual who displayed ATRA resistance (Lanotte et al., 1991; Roussel and Lanotte, 2001; Sun et al., 2004). We cultured NB4 and AP-1060 cells in media supplemented with ATRA in concentrations ranging from 10 nM to 10 μM and subsequently evaluated their propensity to differentiate (Figure 1E; see STAR Methods). Using flow cytometry to measure expression of CD11b and CD18, two integrins expressed by mature innate immune cells (May and Machesky, 2001), we confirmed that NB4 cells respond to ATRA, whereas AP-1060 cells were largely resistant (Figure S2).

**Figure 2:**
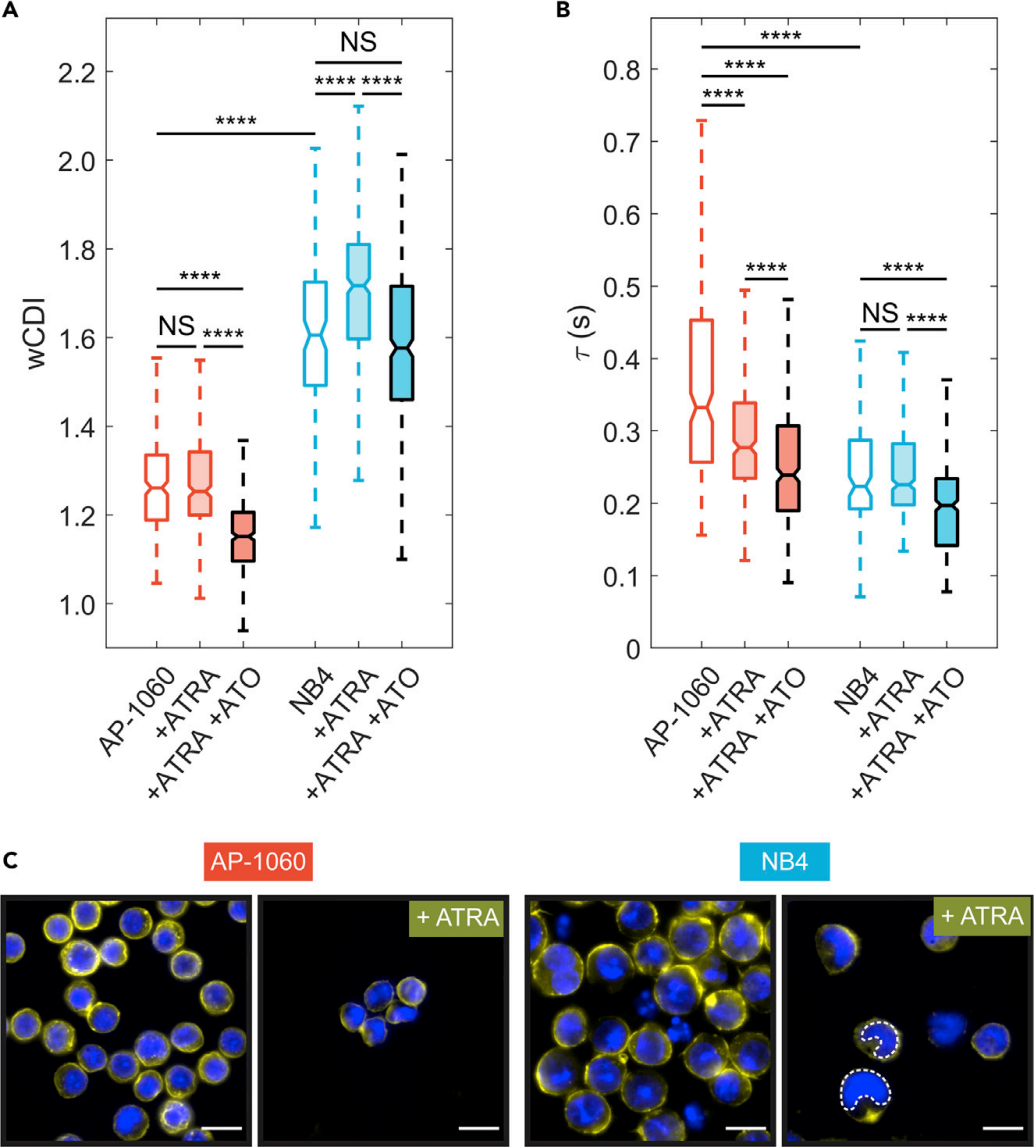
APL cell responses to all-trans retinoic acid (A and B) Box plots of whole-cell deformability index (*wCDI*) (A) and recovery times (B) for AP-1060 and NB4 cells before and after treatment with all-trans retinoic acid (ATRA) and arsenic trioxide (ATO). Notches represent 95% confidence intervals for the true median of each distribution. AP-1060 n = 246; AP-1060 + ATRA n = 333; AP-1060 + ATRA + ATO n = 424; NB4 n = 124; NB4 + ATRA n = 402; NB4 + ATRA + ATO n = 419 from three biological replicates, each measured on a different device. ∗∗∗∗p < 0.0001, NS corresponds to no significance; statistical significance was determined by a Tukey test for multiple comparisons. (C) Fluorescence images of AP-1060 and NB4 cells before and after treatment with ATRA. Cell nuclei were stained with Hoescht 33342 (blue); F-actin was stained with rhodamine phalloidin (yellow). Lobulated (U-shaped) nuclei in ATRA-treated NB4 cells are highlighted with a white dashed outline. Scale bar, 15 μm. See also Figures S2–S4, and Table S1

The differences between AP-1060 and NB4 cells extend well beyond their biochemical response to ATRA. Upon mechanophenotyping these cells, we found that untreated AP-1060 cells have a significantly lower *wCDI* (p < 0.0001) than that of untreated NB4 cells, indicating that they are stiffer (Figure 2A). After treating both cell lines with two doses of 1 μM ATRA over the course of 4 days (see STAR Methods), we observed that only NB4 cells showed a significant change in their *wCDI* (p < 0.0001) and became even more deformable (Figure 2A). Although the ATRA dose we chose did lead to a limited degree of maturation in AP-1060 cells (Figure S2C), there was still no significant change in their overall deformability.

As with their *wCDI*s, we observed differences between the apparent viscosities of the two cell lines via their recovery times. AP-1060 cells were more viscous, taking a far longer time to recover from deformation than NB4 cells (p < 0.0001) (Figure 2B). Although ATRA-treated AP-1060 recovered significantly faster from deformation than untreated AP-1060 cells (p < 0.0001), we surprisingly observed no significant difference between the recovery times of ATRA-treated and untreated NB4 cells (p = 0.99). Although AP-1060 cells are overall resistant to ATRA according to their expression of CD11b and CD18, these cells are not completely unresponsive to ATRA when introduced to a high concentration, as has been previously shown (Figure S2) (Sun et al., 2004). This partial response, as we have discovered, also corresponds to a partial response in mechanical phenotype where changes in recovery time, but not *wCDI,* occur. Mechano-NPS analysis of ATRA-treated APL cells sorted for CD11b expression showed that both CD11b+ AP-1060 and NB4 cells were far more deformable than CD11b- AP1060 cells (p < 0.0001) (Figure S2D) and that only for AP-1060 did CD11b+ cells recover significantly faster than their CD11b- counterparts (p < 0.0001) (Figure S2E). Together, these results show that the differences in responses to ATRA between AP-1060s and NB4s affect not only protein expression but also mechanical phenotype, in which ATRA treatment promotes cell softening in cells that are induced to differentiate.

We additionally evaluated whether arsenic trioxide (ATO), which is commonly administered in conjunction with ATRA to prevent relapse (Adès et al., 2018), affects APL cell mechanics (Figure 1E). Mechano-NPS analysis of AP-1060 and NB4 cells treated with ATRA with 0.25 μM ATO showed that these cells were significantly less deformable and faster to recover compared with those only treated with ATRA (p < 0.0001) (Figures 2A and 2B). ATO is known to alleviate APL primarily by promoting degradation of the PML-RARα fusion protein, whereas ATRA-induced differentiation involves transcriptional regulation mediated by retinoid signaling pathways (Geoffroy et al., 2010; Tatham et al., 2001; Tomita et al., 2013). We hypothesize that it is this difference in pharmacological mechanisms that is reflected in our observations with mechano-NPS; i.e., the cell stiffening after ATO treatment relates to the fact that ATO does not induce differentiation like ATRA.

Because the spatial organization and reorganization of the nucleus constitute a critical part of cell mechanics (Dahl et al., 2008; Friedl et al., 2011; Wolf et al., 2013), we employed fluorescence microscopy to image the nuclei of APL cells before and after ATRA treatment (see STAR Methods). We observed lobulation only in NB4 nuclei (recognizable by the U-shaped nuclei) after ATRA treatment, indicating that differentiation in promyelocytes was progressing (Figure 2C) (Lawrence et al., 2018). In contrast, we did not observe such lobulation in ATRA-treated AP-1060 nuclei, which is consistent with the resistance of these cells to differentiate. In general, lobulation is associated with downregulation of lamin A, a protein associated with the nuclear lamina that strongly influences nuclear and whole-cell deformability (Lammerding et al., 2004; Rowat et al., 2013). Although immunoblotting for lamin A revealed no detectable expression of this protein in either APL cell line both before or after ATRA treatment (Figure S3), this result was expected given prior western blotting of HL-60 (Rowat et al., 2013), a cell line that was derived from a patient mistakenly diagnosed with APL but later found to lack the PML-RARA fusion gene (Dalton et al., 1988). Despite the lack of a detectable amount of lamin A in our samples, the striking morphological changes in NB4 nuclei nevertheless suggest that ATRA-associated softening is strongly influenced by structural changes in the nucleus, although such changes have also been shown to rely on cytoskeletal factors (Olins and Olins, 2004).

We examined the F-actin network, in addition to the nucleus, which provides mechanical support for the cell and its organelles and drives cell motility (Chalut and Paluch, 2016; Gardel et al., 2010; Pegoraro et al., 2017; Pollard and Cooper, 2009; Yamaguchi and Condeelis, 2007). Although there were no major visual differences in the F-actin network between cell types or before and after ATRA treatment (Figure 2C), we sought to determine how F-actin might affect cell mechanical properties. We destabilized F-actin with Latrunculin A (LatA) (see STAR Methods) and confirmed the disruption of cortex-localized F-actin in both AP-1060 and NB4 cells (Figure S4A). Upon mechano-NPS screening of LatA-treated APL cells, we observed no significant differences in the deformability of AP-1060 (p = 0.61) or NB4 (p = 0.19) cells (Figure S4B). However, the recovery times for both cells with disrupted actin cortices were significantly faster than those of untreated cells (AP-1060, p < 0.0001; NB4, p = 0.0027) (Figure S4C), with differences more pronounced in AP-1060 cells than in NB4 cells (125 versus 27 ms faster median recovery, respectively). These results agree with previous work in the literature utilizing latrunculins to measure the mechanical properties of cells in microfluidic constrictions (Byun et al., 2013; Kim et al., 2018). Specifically, it was shown that actin destabilization had a greater effect on the viscoelastic processes of cells undergoing deformation (e.g., the time needed for a cell to deform and enter a constriction, or to recover from deformation) than on the transit velocity through narrow constrictions (Byun et al., 2013; Kim et al., 2018). Overall, since ATRA affected both the elastic and viscoelastic regimes of APL cells and LatA only affected cell viscoelasticity, we conclude that the mechanical changes in ATRA-treated APL cells are not solely due to any remodeling of the F-actin network that may arise after ATRA treatment.

### APL single-cell mechanical phenotypes vary with DNA content

**Figure 3:**
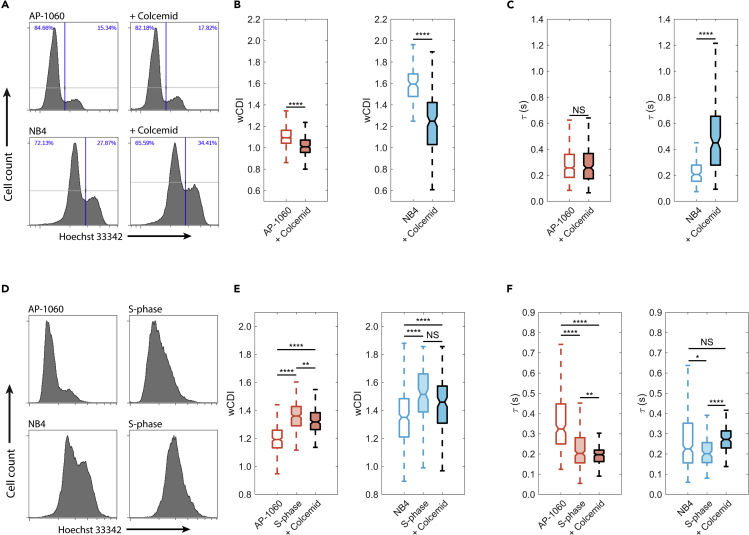
Mechanical phenotypes of APL cells are strongly influenced by cell cycle phase (A) Histograms of DNA content in AP-1060 and NB4 cells before and after colcemid treatment. Colcemid treatment caused small increases in cell counts staining high for DNA, indicating an increased mitotic index. (B and C) Box plots of *wCDI* (B) and recovery times (C) for AP-1060 and NB4 cells before and after treatment with colcemid. Notches represent 95% confidence intervals for the true median of each distribution. AP-1060 n = 616; AP-1060 + Colcemid n = 236; NB4 n = 123; NB4 + Colcemid n = 167 from three biological replicates, each measured on a different device. ∗∗∗∗p < 0.0001, NS, no significance; statistical significance was determined by two-sample Student’s t tests. (D) Histograms of DNA content in AP-1060 and NB4 cells before and after synchronization at S-phase with a double thymidine block. Cell cycle synchronization reduces the bimodal distribution to a unimodal distribution, representing a decreased mitotic index. (E and F) Box plots of *wCDI* distributions (E) and recovery times (F) for AP-1060 and NB4 cells before and after double thymidine block. Notches represent 95% confidence intervals for the true median of each distribution. AP-1060 n = 268; AP-1060 S-phase n = 282; AP-1060 + Colcemid n = 209; NB4 n = 341; NB4 S-phase n = 122; NB4 + Colcemid n = 151 from three biological replicates, each measured on a different device. ∗p < 0.05, ∗∗p < 0.01, ∗∗∗∗p < 0.0001, NS, no significance; statistical significance was determined by two-sample Student’s t tests. See also Figure S5 and Table S1

Because DNA content and nuclear volume change throughout a cell’s life cycle, cell cycle phase is also an important contributor to whole-cell mechanical properties (Aureille et al., 2019; Chu et al., 2017; Webster et al., 2009). We demonstrated this by performing a series of experiments in which we mechanically phenotyped cells that were synchronized in either M- or S-phase (high or low DNA content, respectively). Specifically, we used colcemid (a mitotic inhibitor) to arrest cells in M-phase prior to mechanophenotyping (Figures 1F and 3A; see STAR Methods). Both NB4 and AP-1060 cells showed a significant increase in stiffness (p < 0.0001), with stiffening more pronounced in the former cells (Figure 3B). Only colcemid-treated NB4 cells had significantly slower recovery times, and thus were more viscous, as compared with their respective untreated cells (p < 0.0001) (Figure 3C).

To determine whether low DNA content would lead to increased deformability and lower viscosity, we mechanophenotyped AP-1060 and NB4 cells that we arrested in S-phase using a double thymidine block, which prevents DNA synthesis (Figures 1F and 3D) (Bostock, 1971). In contrast to cells arrested in M-phase, cells in S-phase were significantly more deformable than untreated cells (p < 0.0001) (Figure 3E). Moreover, compared with non-arrested cells, both NB4 and AP-1060 cells recovered from deformation significantly faster after the double thymidine block (NB4, p = 0.0105, AP-1060, p < 0.0001) (Figure 3F).

Thus, increasing or decreasing the DNA content can also increase or decrease cell stiffness. To further confirm that DNA content is a strong driver of whole-cell mechanical phenotype, we mechanophenotyped NB4 cells that we sorted based on DNA content (see STAR Methods) and subsequently and similarly observed that low-DNA-content cells had a greater *wCDI* and faster recovery time compared with high-DNA-content cells (Figure S5).

Because it inhibits cell division by interfering with microtubule dynamics, we next focused on determining colcemid’s contribution to cell mechanical phenotype independent of modulating DNA content. We first used a double thymidine block to arrest AP-1060 and NB4 cells in S-phase and then treated these cells with colcemid. The *wCDI* was significantly smaller after this treatment for AP-1060 cells only (p < 0.01) (Figure 3E). However, the recovery time of both AP-1060 and NB4 cells significantly increased (p < 0.01 and p < 0.0001, respectively) (Figure 3F). Thus, colcemid does affect the mechanical phenotype of AP-1060 and NB4 cells, although these effects are far less dramatic than the cell stiffening we observed when cells were arrested in M-phase.

### Influences of histone acetylation on APL cell mechanical phenotype

We next probed how the mechanical phenotypes of APL cells depend on the modulation of chromatin accessibility downstream of retinoid signaling (e.g., due to ATRA) (Bernardi and Pandolfi, 2007; Mendez et al., 2018; Nervi et al., 1998). We perturbed chromatin accessibility independently of ATRA treatment by inhibiting histone deacetylase (HDAC) activity with trichostatin A (TSA) (Figure 1G; see STAR Methods), which has been previously shown to soften isolated nuclei (Stephens et al., 2017, 2018). To measure primarily the mechanical properties of the nucleus and the effect of TSA, we destabilized actin cortices with LatA and arrested cells in S-phase using a double thymidine block (see STAR Methods). We then treated the cells with TSA or ATRA and subsequently mechanophenotyped them. As a control, we mechanophenotyped synchronized and LatA-treated cells without further perturbations.

**Figure 4:**
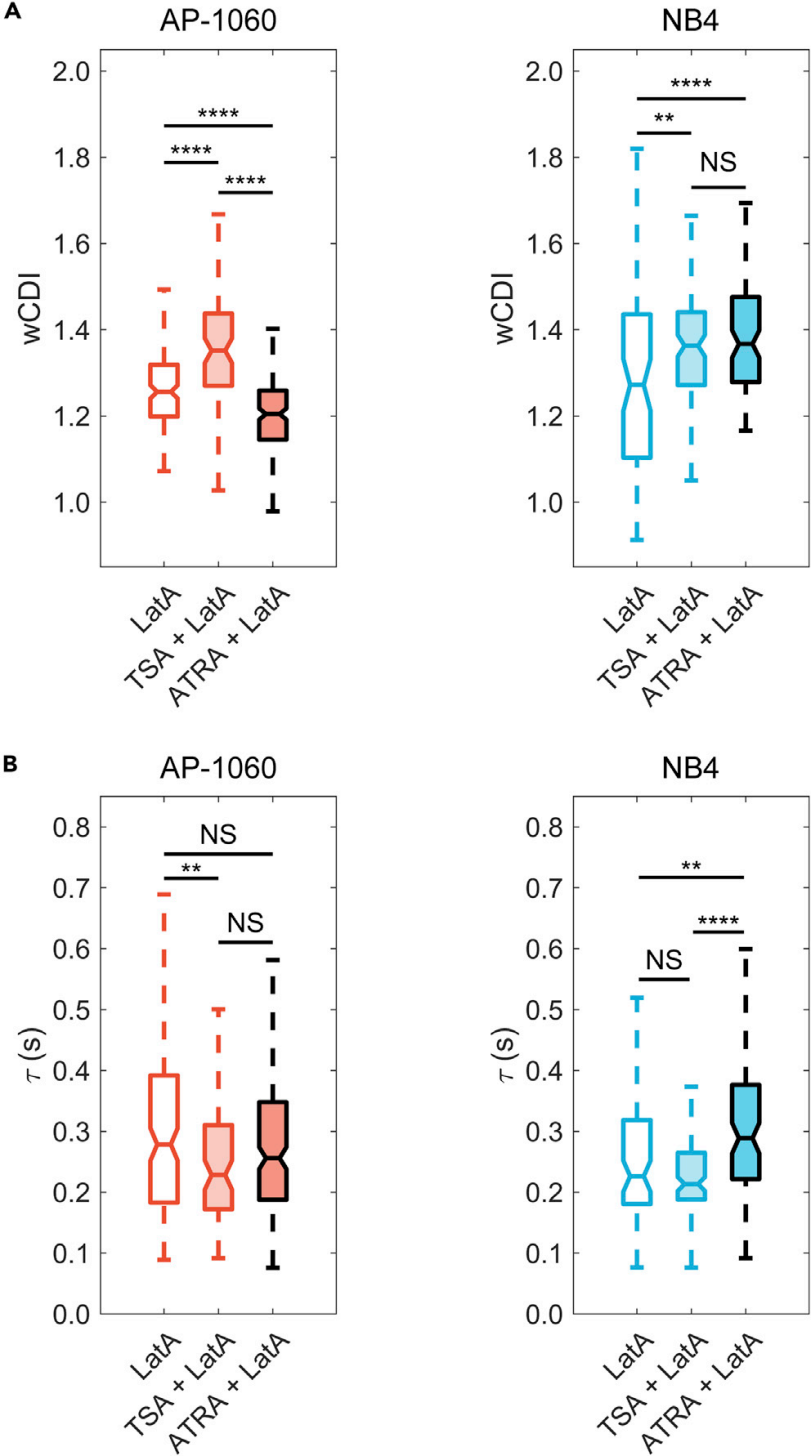
Mechanical phenotype of ATRA-resistant APL nuclei is sensitive to histone acetylation (A and B**)** Box plots of *wCDI* (A) and recovery times (B) for S-phase synchronized AP-1060 (left) and NB4 (right) with actin cortices destabilized with Latrunculin A (LatA). Cells were treated either with trichostatin A (TSA) to promote accumulation of decondensed chromatin or with ATRA to induce differentiation. AP-1060 + LatA n = 155; AP-1060 + TSA + LatA n = 84; AP-1060 + ATRA + LatA n = 199; NB4 + LatA n = 75; NB4 + TSA + LatA n = 114; NB4 + ATRA + LatA n = 95 from three biological replicates, each measured on a different device. ∗∗p < 0.01, ∗∗∗∗p < 0.0001, NS, no significance; statistical significance was determined by a Tukey test for multiple comparisons. See also Table S1

TSA treatment significantly increased *wCDI* in both AP-1060 (p < 0.0001) and NB4 (p = 0.0014) cells, suggesting that chromatin decondensation indeed causes nuclear softening (Figure 4A). In contrast to our earlier experiments when cells were solely treated with ATRA (Figure 2A), we unexpectedly observed a dramatic decrease in *wCDI* (p < 0.0001) of ATRA-treated, actin-destabilized, and S-phase arrested AP-1060 cells (Figure 2A). Moreover, actin-destabilized and S-phase-arrested NB4 cells were more deformable after ATRA treatment (p < 0.0001) (Figure 4A, right), agreeing with the results of our earlier experiments when these cells were only treated with ATRA (Figure 2A). Recovery time responses were also different depending on treatment: TSA significantly decreased recovery times in AP-1060 (p = 0.0095) but not NB4 (p = 0.13) cells (Figure 4B); ATRA-treated AP-1060 cells showed no substantial difference in recovery time (p = 0.15); but NB4 cells took far longer to recover (p = 0.0030).

Overall, these measurements highlight the complexity of measuring whole-cell deformability, where the effects of certain perturbations (i.e., ATRA) on cell stiffness can change, depending on the state of other subcellular components (i.e., F-actin, DNA content). For actin-destabilized and S-phase-arrested cells, TSA had a more consistent softening effect than ATRA across both cell types and mechanical parameters, most likely because TSA causes chromatin decondensation. Although nuclear softening is expected, TSA treatment may also modulate the expression of genes that could affect single-cell mechanical phenotype, such as those coding for proteins that regulate cytoskeletal protein dynamics (Ceccacci and Minucci, 2016; Minucci et al., 2001).

### Characterizing transcriptomic changes in APL cells with RNA-seq

Studies involving cDNA microarrays have identified patterns of differential gene expression in APL cells undergoing differentiation after ATRA treatment (Lee et al., 2002; Yang et al., 2003). In contrast, total RNA sequencing captures entire transcriptomes and can thus provide a more thorough exploration of how ATRA differentially affects the gene expression of ATRA-responsive and ATRA-resistant cells. Here, we performed RNA-seq on APL cells treated with both ATRA and TSA to identify transcriptional changes potentially associated with mechanical response to treatment. To compare, we performed the same treatments and RNA-seq analysis on HL-60 cells, which we found to lack the same softening response as differentiating APL cells (Figures 2 and S6). We thus performed RNA-seq on these three cell lines under four different conditions and identified differentially expressed genes between samples and conditions (Mccarthy and Smyth, 2009).

In comparing cell lines, NB4 had the most differentially expressed genes (2,523) between untreated and ATRA-treated samples (Figure S7A). AP-1060 had fewer differentially expressed genes (1,907), reflecting its ATRA resistance, and HL-60 had the least (1,176), corresponding to its lack of sensitivity to ATRA due to the absence of the PML-RARA fusion gene. Although NB4 cells demonstrated a stronger response to ATRA than AP-1060, there was only one differentially expressed gene (C1QA, part of the classical complement pathway) between TSA-treated and untreated NB4 versus 86 with AP-1060 in a similar comparison (Figure S7B). We also evaluated the interaction of TSA with ATRA by comparing gene expression of ATRA-treated cells to cells treated with both TSA and ATRA. This analysis revealed 13 differentially expressed genes in NB4, compared with 385 in AP-1060 (Figure S7C). The greater number of differentially expressed genes in AP-1060 compared with NB4 underscores the former’s sensitivity to TSA HDAC inhibition that we previously measured with mechano-NPS.

**Figure 5:**
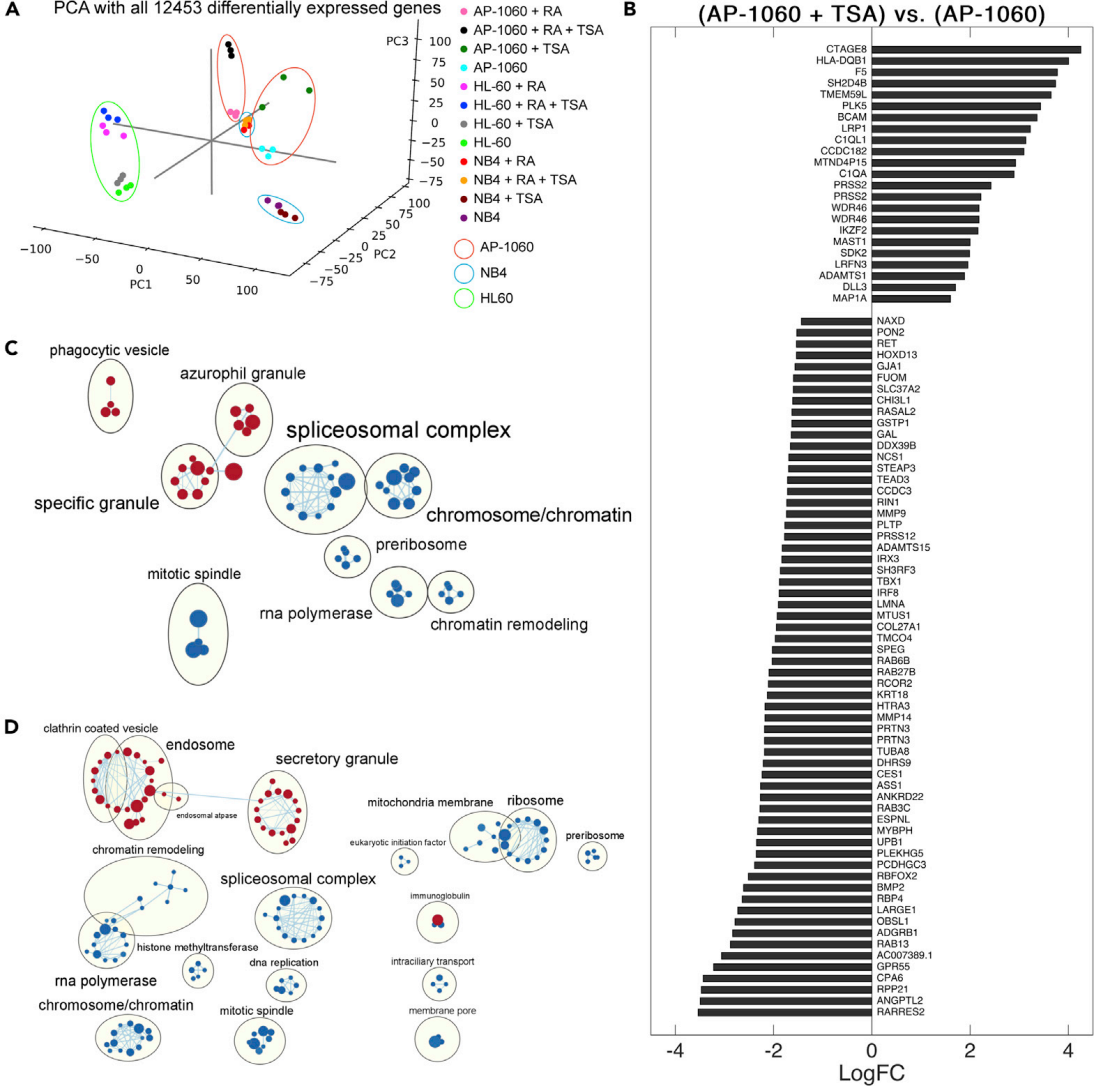
RNA-seq and differential expression analysis of APL cells highlights transcriptional differences in ATRA-resistant AP-1060 cells (A) Principal component analysis (PCA) of counts per differentially expressed gene identified in any of 66 between-sample pairwise comparisons. (B) Differentially expressed genes identified in comparing TSA-treated versus untreated AP-1060. (C) Enrichment map of positively (red) and negatively (blue) enriched gene sets for ATRA-treated AP-1060 cells. Gene set enrichment analysis (GSEA) was performed from the *GO_CC* (Gene ontology cellular component) collection. (D) Enrichment map of positively (red) and negatively (blue) enriched gene sets for ATRA-treated NB4 cells. GSEA was performed from the *GO_CC* (Gene ontology cellular component) collection. See also Figures S6 and S7

We performed principal component analysis on all differentially expressed genes and evaluated the loadings of individual genes to each principal component (Figures 5A and S7D–S7G). The first two principal components (PC1 and PC2) clearly separated the different cell types. The third principal component (PC3) separated only AP-1060 samples treated with TSA. Analyzing the top 100 highest-loading genes for each principal component, PC3 was especially enriched for gene ontology terms associated with immune cell function and activation compared with PC1 and PC2. PC1 was primarily enriched for cellular reorganization and RNA processing. PC2 was enriched for several biological processes including metabolism and cell activation.

We analyzed the expression of the 86 differentially expressed genes between untreated AP-1060 and TSA-treated AP-1060. The retinoid signaling-related genes RARRES2 and RBP4 were among the most highly downregulated genes (1^st^ and 11^th^, respectively) (Figure 5B). RARRES2 was found to be upregulated in an HL-60 population made multi-drug resistant via selection with ATRA, and RBP4 has been shown to promote the extracellular transport of retinoic acid (Blaner, 2019; Liu et al., 2015). As such, downregulation of these two genes could increase sensitivity to ATRA. We filtered these 86 genes by association to the cytoskeleton to identify genes that might also affect cell structure and, by extension, mechanical properties. Several microtubule-associated genes were downregulated, as were cytoskeleton-associated OBSL1, MYBPH, EPSNL, ANKRD22, KRT18, and LMNA (in decreasing order of log-fold change in expression). Only microtubule-associated MAP1A was upregulated. Although the softening in TSA-treated AP-1060 cells is most likely due to direct decondensation of chromatin, the downregulation of these cytoskeletal genes may also contribute to cell softening. Future work is necessary to quantify whether any of these genes, by themselves or in concert, have a substantial impact on mechanical phenotype.

We next investigated the differentially expressed genes in AP-1060 and NB4 after 96 h of ATRA treatment. First, we compared our differential expression results with cDNA microarray results from Yang et al. in which NB4 cells were also treated with ATRA for 96 h. Although we found agreement in 24 of 62 upregulated genes and 15 of 43 downregulated genes (Yang et al., 2003), we did not find the remaining genes to be differentially expressed as Yang et al. had. To understand the whole-transcriptome effects of ATRA on our APL cells, especially in the context of controlling cell mechanical phenotype, we carried out gene set enrichment analysis (GSEA) in order to compare ATRA-treated AP-1060 and NB4 samples with their untreated counterparts. Enrichment maps from this analysis demonstrated a wide range of transcriptional effects from ATRA and additionally reflected the more dramatic response in NB4 (Figures 5C and 5D). ATRA treatment for both cell lines resulted in positive enrichment of gene sets for granulocytic functions (phagocytosis, granules) associated with differentiation induced by ATRA treatment, with this effect being more pronounced in NB4, as expected. Negatively enriched gene sets in both cell lines were linked to transcription, RNA processing, and chromosome/chromatin organization (Figure 5C), reflecting the transcriptional and epigenetic regulation that occurs downstream of retinoid signaling (Bernardi and Pandolfi, 2007; Mendez et al., 2018; Nervi et al., 1998). Furthermore, ATRA-treated NB4 were also negatively enriched for components of DNA replication and epigenetic regulation, including histone methyltransferases (Figure 5D). As we showed that DNA content (Figure 3) and chromatin decondensation (Figure 4) both modulate mechanical phenotype, genes affecting the synthesis and organization of DNA may be important drivers of cell softening in ATRA-treated NB4.

Last, we constructed a gene set to investigate specifically the immunophenotypic landscape of differentiating AP-1060 and NB4 cells. As expected, NB4 cells exhibited greater upregulation of innate immunity-related genes (indicative of differentiation) after ATRA treatment compared with AP-1060 (Figure S5H). These data provide a more thorough transcriptomic analysis of ATRA-induced differentiation compared with prior work using cDNA microarrays (Lee et al., 2002; Yang et al., 2003), as well as how this response differs in an ATRA-resistant cell line.

## Discussion

APL is a highly aggressive AML subtype that can quickly lead to patient death if therapeutic ATRA regimens are unsuccessful. ATRA induces differentiation of malignant immature promyelocytes, which would otherwise proliferate to the point of patient morbidity. Using mechano-NPS, we have uncovered specific cell mechanical properties that correlate with ATRA-resistant APL. Specifically, we determined that ATRA-resistant AP-1060 cells are stiffer and more viscous than ATRA-sensitive NB4 cells. Through an in-depth study, we determined that the mechanics of APL cells are more dependent on the mechanical properties of their nuclei rather than cytoskeletal components such as microtubule and actin networks. Specifically, major changes in DNA content strongly influenced cell mechanical phenotypes, with high DNA content associated with stiffer and more viscous cells. Although it had a more subtle effect on APL cells, chromatin decondensation led to the softening of ATRA-resistant AP-1060 cells, a response seen only in differentiating APL cells. Through RNA-seq, we discovered enriched gene sets that may also affect mechanical phenotype by way of chromatin remodeling. We also discovered several cytoskeleton-associated genes that were differentially expressed, and their potential influence on mechanical phenotype should be investigated in future studies. Because ATRA or TSA could directly affect cell structure without affecting transcriptional regulation (e.g., like the destabilization of actin with LatA), it would be valuable to know if changes in transcription, protein structure, or both are what drive changes in mechanical phenotype.

The importance of nuclear mechanics in APL is further strengthened when considering our results with cells that were synchronized into S-phase and had their F-actin networks disrupted. Synchronized and actin-destabilized AP-1060 cells treated with TSA were more deformable and less viscous than similarly treated NB4 cells. However, when synchronized and treated with LatA, AP-1060 cells stiffened after ATRA treatment, an effect not observed with unsynchronized cells with intact actin. This may be a consequence of AP-1060’s ATRA resistance and the unique epigenomic state of S-phase synchronized cells, where decondensed chromatin is more accessible and susceptible to retinoid signaling (Alabert and Groth, 2012). However, further work is warranted to identify the exact mechanism behind this unexpected synergistic effect between cell cycle synchronization and ATRA. Unlike AP-1060 cells, synchronized NB4 cells with disrupted F-actin networks consistently softened after ATRA treatment. Overall, we conclude that NB4 is robustly sensitive to ATRA, whereas AP-1060 is sensitive to HDAC inhibition with TSA.

Identifying the biophysical factors that link a particular pathological phenotype to a specific mechanical phenotype is a new approach toward the implementation of physical and mechanical biomarkers to characterizing cancer cells. Understanding these factors can explain the biological underpinnings of the mechanical properties of cancer cells, whose potential clinical applications are frequently discussed (Carey et al., 2019; Kozminsky and Sohn, 2020). Within this light, we systematically perturbed subcellular components of APL cells and assessed their relevance to the mechanical phenotype associated with ATRA resistance. In our RNA-seq analysis, we highlighted genes and gene sets associated with our observations of APL cells and their mechanical properties and provided a resource characterizing the transcriptome of differentiating and ATRA-resistant APL cells. We bridge together two critically under-examined aspects of APL, ultimately uncovering a previously unknown relationship between drug resistance and mechanical phenotype.

### Limitations of the study

Our study and the results therein have certain limitations. First, because so few APL cell lines are available, we were only able to study NB4 and AP-1060 cells as examples of ATRA-responsive and ATRA-resistant cell lines, respectively. Studying primary cultures with differing responses to ATRA would provide a more thorough perspective on how APL cell mechanics are related to ATRA sensitivity. Second, we observed several important effects of TSA treatment with RNA-seq; however, this method does not directly measure changes in chromatin condensation and accessibility. Assays such as ATAC-seq or ChIP-seq could confirm whether TSA treatment decondenses chromatin and whether, for example, differentially expressed genes like RARRES2 and RBP4 in TSA-treated AP-1060 are also differentially accessible. Third, although we identified several gene sets and differentially expressed genes that may link the mechanical phenotypes of APL cells to their responses to ATRA, direct perturbations of these genes or their gene products could provide further elucidation regarding how ATRA treatment influences APL cell mechanics. Fourth, although we hypothesize that the differential expression of genes and gene products are the main drivers of cell mechanical properties, we note that we did not account for the possibility that these pharmacological agents could directly affect cell mechanics without affecting protein expression (e.g., direct conformational changes in proteins already abundant in the cell). This could be controlled for by inhibiting translation, such as with cycloheximide. Fifth, mechano-NPS applies a significant deformation to cell nuclei and as a result, the whole-cell mechanical phenotype may overestimate the mechanical contributions of the nucleus and underestimate those of cytosol and membrane-associated subcellular components. As such, some of the observed mechanical behavior may be less apparent if different techniques are used, such as those that apply a tensile strain (e.g., optical stretchers or micropipette aspiration here) (Guck et al., 2005; Hochmuth, 2000) instead of the compressive strain used. In addition, although *wCDI* accounts for differences in overall cell diameter, certain perturbations we made (such as those controlling DNA content) may cause changes in nuclear volume, affecting the strain applied to the nucleus and altering the deformability of the whole cell.

## STAR★Methods

### Key resources table

**Table:** 

REAGENT or RESOURCE	SOURCE	IDENTIFIER
**Antibodies**		

FITC-anti-CD11b	Biolegend	Cat#101206; RRID: AB_312789
Super Bright 600-anti-CD18	Invitrogen	Cat#63-0189-41; RRID: AB_2734896
anti-Lamin A/C	Invitrogen	Cat#MA1-06101; RRID: AB_559889
anti-GAPDH	CellSignaling	Cat#2118S; RRID: AB_561053
IRDye® 680RD Goat anti-Mouse IgG Secondary Antibody	LI-COR	Cat#926-68070; RRID: AB_10956588
IRDye® 800CW Goat anti-Rabbit IgG Secondary Antibody	LI-COR	Cat#926-32211; RRID: AB_621843

**Chemicals, peptides, and recombinant proteins**		

All-trans retinoic acid	Sigma-Aldrich	Cat#R2625
Arsenic trioxide	Sigma-Aldrich	Cat#202673
Colcemid	Gibco	Cat#15212012
Latrunculin A	Abcam	Cat#ab144290
Thymidine	Abcam	Cat#ab143719
Trichostatin A	Fisher Scientific	Cat#14-061

**Deposited data**		

RNA-Seq data	This paper	NCBI GEO: GSE175507

**Experimental models: Cell lines**		

AP-1060	Dr. Scott Kogan, UCSF	RRID:CVCL_1807
5637	ATCC	RRID:CVCL_0126
NB4	DSMZ	RRID:CVCL_0005
HL-60	ATCC	RRID:CVCL_0002
MDA-MB-231	University of California, Berkeley Cell Culture Facility	RRID:CVCL_0062

**Software and algorithms**		

MATLAB	Mathworks	RRID:SCR_001622
ImageJ	NIH	RRID:SCR_003070
Cytobank	Beckman Coulter	RRID:SCR_014043
Mechano-NPS algorithms	This paper	https://github.com/sohnlab/mechanoNPS_Li-et-al-2020/releases/tag/mNPS_2020

### Resource availability

#### Lead contact

Further information and requests for resources and reagents should be directed to and will be fulfilled by the lead contact, Prof. Lydia L. Sohn (sohn@berkeley.edu)

#### Materials availability

This study did not generate new unique reagents.

### Experimental model and subject details

AP-1060 cells (sex: male), obtained from Dr. S. Kogan, University of California-San Francisco, San Francisco, CA, U.S.A., were cultured in 70% IMDM (Gibco 12440053) supplemented with 20% FBS, 1% Penicillin-Streptomycin (Gibco 15070063), and 10% conditioned medium from cell line 5637 (ATCC HTB-9). For conditioned medium from cell line 5637, 2.5 x 10^6^ cells were seeded at in 10 mL of RPMI-1640 (Corning 10-040-CV) supplemented with 10% FBS and 1% Penicillin-Streptomycin (Gibco 15070063). Medium was exchanged after 24 hours, then collected at 48 hours after initial seeding. Conditioned medium was then sterilized using a 0.22 μm polyethylsufone filter (Millipore Sigma SLGPM33RS). NB4 (DSMZ ACC 207) and HL-60 (ATCC CCL-240) cells were cultured in 90% RPMI-1640 supplemented with 10% FBS and 1% Penicillin-Streptomycin. AP-1060 cells were passaged at a density of 2 x 10^6^ cells/mL, seeded in wells of a 24-well plate at a density of 1 x 10^6^, and maintained at 37°C in 5% CO_2_. NB4 (sex: female) and HL-60 (sex: female) cells were passaged at a density of 1 x 10^6^ cells/mL, seeded in wells of a 24-well plate at a density of 5 x 10^5^, and maintained at 37°C in 5% CO_2_. MDA-MB-231 cells (sex: female) were obtained from the University of California, Berkeley Cell Culture Facility, and were cultured in DMEM with 10% FBS and maintained at 37 °C with 5% CO_2_. MDA-MB-231 cells were passaged at 75% confluence and seeded at 1 x 10^6^ cells in 100 mm polystyrene dishes (ThermoScientific 12-567-650). To passage MDA-MB-231 cells, dissociation was accomplished by incubating cells in 0.25% trypsin-EDTA (Gibco, 25200056) at 37 °C and 5% CO_2_ for 5 min, followed by trypsin neutralization with complete DMEM (twice the volume of trypsin solution used). We did not perform cell line authentication for our studies.

### Method details

#### Device design and fabrication

All mechano-NPS devices utilized in these studies consisted of a 12.9 ± 0.1 μm high microfluidic channel molded into a polydimethylsiloxane (PDMS) slab that was bonded to a glass substrate with pre-defined platinum (Pt) electrodes and gold (Au) contact pads. PDMS slabs included in-line filters with a pore size of 20 μm to prevent cell clusters that would clog the channel. The central contraction segment, 7 μm x 2000 μm (W x L), was flanked by a single node-segment at its entrance and 10 recovery segments at its exit. While all nodes were 85 μm x 50 μm (W x L), the single segment located in front of the contraction segment was 13 μm x 800 μm (W x L) and the recovery segments were each 13 μm x 290 μm (W x L). Contraction segment length was chosen such that cells experienced deformation for ∼150-200 ms. The series of 10 recovery segments provided up to ∼500 ms of sampling for recovering cells, which was sufficient to observe the exponential decay of cell strain to a steady-state value.

A standard soft lithography process was used to create the PDMS microfluidic channels. Briefly, negative relief structures were fabricated onto polished silicon wafer using SU8-3010 epoxy resist (MicroChem). SU-8 3010 was spun at 1850 rpm for 30 seconds and baked at 95°C for 8 minutes. The resist-coated wafer was then exposed to a mask with UV light at a dose of 160 mJ/cm^2^, baked again at 65°C for 1 minute, and then at 95°C for 3 minutes. Finally, the wafer was immersed in SU-8 developer (MicroChem) for 2 minutes, then rinsed with water and dried. This process yielded a film thickness and microchannel height of 12.9 ± 0.1 μm (mean ± std. dev.). Sylgard 184 PDMS (Dow Corning) pre-polymer and curing agent were mixed in a ratio of 9:1, degassed, and then poured onto the negative-relief masters. After curing for 2 hours at 85°C, PDMS slabs with the embedded microfluidic channels were cut and peeled from the relief masters, cored with a 1.5 mm biopsy punch (Harris Uni-Core, Fisher Scientific) to provide input/output access to the microchannel, and then cleaned with isopropanol and deionized water (DI, 18 MW).

Pt/Au electrodes were fabricated using a lift-off process with electron-gun evaporation for metal deposition. The Pt electrodes and Au contact pads were patterned onto glass slides using standard photolithography. First, glass slides were patterned with positive-tone S1813 resist (MicroChem) by spin-coating at 3000 rpm and soft-baking at 100°C for 1 minute. The wafer was then exposed to a mask with UV light at a dose of 300 mJ/cm^2^, and subsequently developed in MF-321 developer (MicroChem) for 45 seconds. A thin metal film consisting of 75 Å Ti, 250 Å Pt, and 250 Å Au was then deposited onto the patterned glass slides using electron-gun evaporation. Excess metal and photoresist were lifted off using acetone. The fabricated metal electrodes were then cleaned with acetone, isopropanol, and DI water. The glass slides with pre-fabricated electrodes and the molded PDMS devices were simultaneously treated with oxygen plasma (2 minutes, 450 mTorr, 30 W, Harrick Plasma) before being bonded together and baked at 125°C for 5 minutes.

#### Mechano-node pore sensing

We employed mechano-node pore sensing (mechano-NPS) by performing a four-terminal measurement to measure cells as they transit mechano-NPS channels. (Kim et al., 2018). A DC potential (< 3 V) is first applied across the mechano-NPS channel so that cells transiting the channel partially block the flow of electric current, leading to a modulated current pulse. In the experiments performed, cell suspensions were prepared at a concentration of 300,000 cells/mL in 1X phosphate buffered saline (PBS) solution supplemented with 2% fetal bovine serum (FBS, VWR 89510-186) to reduce cell-cell and cell-PDMS adhesion. Cell suspensions were injected into mechanoNPS devices using a microfluidic pressure controller (Elveflow OB1) with a nominal inlet pressure of 80 mbar. Signals were sampled at 50 kHz, post-processed with a moving-average low pass filter, and then downsampled to 2.5 kHz. A custom command-line interface program written in MATLAB (available on GitHub) rapidly identified mechano-NPS pulses, determined subpulse features (i.e. magnitude and duration), and extracted physical and mechanical parameters.

#### Quantifying rates of cell recovery from deformation

The cell can be approximated as a homogeneous sphere of a Kelvin-Voigt material. Its release from the constriction channel into recovery segments constitutes a step change in applied compressive stress. Consequently, the cell strain will relax to a steady-state value according to the following exponential decay model:

(Equation 3) $$\varepsilon \left(t\right)=\varepsilon_{0}\;\exp\left(-t/\tau \right)+\varepsilon_{\infty }$$

where *ε*(*t*) is the strain over time after the step change in stress, *ε*_0_ is the strain at the time of the step change in stress, τ is the time constant of the exponential decay, and *ε*_∞_ is the steady-state value for strain after the cell has fully relaxed. In node-pore sensing techniques, the magnitude of the initial current drop is related to the size of the particle (DeBlois and Bean, 1970; Saleh and Sohn, 2001). A transform of variables from the stress-strain space to the voltage-current space gives the following equation governing the measurement of this viscoelastic creep in mechano-NPS:

(Equation 4) $$\text{Δ}I\left(t\right)=\text{Δ}I_{0}\left[1-\exp\left(-t/\tau \right)\right]+\text{Δ}I_{\infty }$$

where Δ*I* is equivalent to a difference in current from the baseline: *I*_*baseline*_−*I*(*t*), Δ*I*_0_ is the current drop associated with a cell at the time of release from the constriction channel, and Δ*I*_∞_ is the current drop associated with a cell at its steady-state strain after complete relaxation. Rewriting Equation (4) as a function of measured current rather than current drop yields:

(Equation 5) $$I\left(t\right)=I_{0}\text{ exp}\left(-\text{t}/\text{τ}\right)+I_{\infty }$$

where *I*(*t*) is the measured current over time after the step change in stress, *I*_0_ is the current associated with the cell at the time of release from the constriction channel, *τ* is the time constant of the exponential decay in voltage, and *I*_∞_ is the current associated with the cell at its steady-state strain after complete relaxation. Mechano-NPS subpulses produced after the cell exits the constriction channel provide values for *I*(*t*) and *t* to which we fit a linear model to estimate *τ*. To perform linear least squares regression for this model, we linearize Equation (5) by subtracting *I*_∞_ from *I*(*t*) and taking the logarithm of both sides:

(Equation 6) $$\ln\left[I\left(t\right)-I_{\infty }\right]=\ln\;I_{0}-t∕\tau$$

After performing linear least squares, we can then use the slope of the linear model to calculate a confidence interval for the time constant of the exponential decay, thus estimating a cell’s rate of recovery from deformation.

#### Pharmacological treatments

Dry powder all-trans retinoic acid (ATRA, Sigma-Aldrich R2625) was reconstituted in anhydrous dimethylsulfoxide to 25 mg/mL for an 83.3 mM concentrated stock solution (Sigma-Aldrich 276855). Concentrated stock solution was aliquoted and snap-frozen in liquid nitrogen, then transferred to a -80°C freezer for long-term storage. Concentrated stock ATRA was thawed and diluted to 10 mM in dimethyl sulfoxide (DMSO) prior to use, then further diluted to working concentration (10 nM to 10 μM for testing ATRA response; 1 μM otherwise) in cell-culture media. Dry powder arsenic trioxide (ATO, Sigma-Aldrich 202673) was prepared in a 2 mM solution by first dissolving dry ATO in 10% w/w sodium hydroxide (NaOH) in DI water. The NaOH was then neutralized with an equimolar amount of 1 M hydrochloric acid, then diluted with additional DI water. Concentrated stock ATO was diluted to 25 μM in DI water prior to use, then further diluted to 0.25 μM in cell-culture media. Cells receiving both ATRA and ATO received doses of 1 μM ATRA and 0.25 μM ATO upon seeding and at 48 hours after seeding. LatrunculinA (LatA, Abcam ab144290) was reconstituted in ethyl alcohol (Sigma-Aldrich 459844) and added to cell-culture media at a concentration of 0.5 μg/mL for 30 minutes to disrupt actin filaments. 100 nM of Trichostatin A (TSA, Fisher Scientific 14-061, Mfg. Tocris Bioscience), reconstituted in DMSO, was added to cells for 24 hours to inhibit histone deacetylase activity. For synchronized and LatA-treated cells, cell cultures were first synchronized in S-phase (see below). Cells not receiving further treatment were treated with LatA (see above) immediately prior to mechano-NPS analysis. Cells treated with TSA were incubated in cell culture media supplemented with 100 nM TSA for 24 hours, then treated with LatA (see above) immediately prior to mechano-NPS analysis. Cells treated with ATRA were incubated in cell-culture media supplemented with 1 μM ATRA at 0 and 48 hours after the completion of cell-cycle synchronization, then treated with LatA (see above) 96 hours after synchronization, followed by immediate analysis with mechano-NPS. For all pharmacological treatments, cells were pelleted via centrifugation at 200 rcf for 5 minutes, rinsed once with 1X PBS, then pelleted again, and resuspended for mechano-NPS analysis (see above).

#### Cell-cycle arrest

A double thymidine block was used to arrest cells at S-phase. Cells were resuspended at 1 x 10^6^ cells/mL in cell-culture media supplemented with 2 mM thymidine (Abcam ab143719) and incubated at 37°C in 5% CO_2_ for 18 hours. They were then isolated via centrifugation at 200 rcf for 5 minutes, resuspended at the same density in cell-culture media supplemented with 10 μM deoxycytidine (Abcam ab146218), and subsequently incubated at 37°C in 5% CO_2_ for 8 hours. Finally, cells were again collected via centrifugation at 200 rcf for 5 minutes, resuspended at the same density in cell-culture media supplemented with 2 mM thymidine, and incubated at 37°C in 5% CO_2_ for 18 hours. Synchronized cells were then isolated via centrifugation at 200 rcf for 5 minutes and rinsed once with 1X PBS for further experiments. Colcemid solution in 1X PBS (Gibco 15212012) was used to arrest cell division and synchronize cells in M-phase. Colcemid was added directly to cells in culture media at a concentration of 1 μg/mL. Cell cultures were then incubated at 37°C in 5% CO_2_ for 2 hours. Synchronized cells were then isolated via centrifugation at 200 rcf for 5 minutes and rinsed once with 1X PBS for further experiments. Cell cycle arrest in either S- or M-phase was confirmed via flow cytometric analysis of DNA content (see below).

#### Fluorescence staining and immunostaining

Silicone isolators (Grace BioLabs CWS-13R-0.5) were pressed onto poly-L-lysine glass slides (VWR 16002-116) to form small wells. Cells in suspension culture were resuspended at 5 x 10^5^ cells/mL in 1X PBS, then pipetted into the wells, and allowed to settle and stick to the positively charged substrate for 1 hour while incubating at 37°C in 5% CO_2_. Wells were then washed with 1X PBS to remove unbound cells. Cells were then fixed with 4% paraformaldehyde (Sigma-Aldrich P6148) and permeabilized with 0.1% Triton X-100 (Sigma-Aldrich T8787). Fluorescence staining solutions for ATRA and LatA experiments contained the same concentrations of Hoechst 33342 and rhodamine phalloidin.

#### Flow cytometric analysis

Cells were isolated by centrifugation at 200 rcf for 5 minutes and resuspended in 1X PBS. For assessing DNA content, cells were stained with 40 μM Hoechst 33342 for 30 minutes, washed with 1X PBS, and analyzed on a BD LSR Fortessa X20 with BD FACSDiva 9.0 software. For immunostained flow cytometry, cells were blocked with an anti-Fc Receptor polyclonal antibody (Invitrogen 14-9161-73) for 30 minutes. An antibody mix for a three-color flow cytometry panel was prepared, consisting of FITC-anti-CD11b (Biolegend 101206), Super Bright 600-anti-CD18 (Invitrogen 63-0189-41), and PerCP-eFluor 710-anti-CD52 (Invitrogen 46-0529-41). Samples were incubated with fluorophore-conjugated antibodies for 35 minutes, then washed several times with 1X PBS. Samples were then stained with LIVE/DEAD Fixable Violet (Invitrogen L34955) and washed several times with 1X PBS prior to analysis. For each experimental condition, one unstained control and three fluorescence-minus-one controls for each antibody-conjugated fluorophore were prepared (Figure S2). Single-stain positive control samples for each fluorophore were prepared prior to all analysis to compute a compensation matrix. Compensation was set such that the median fluorescence intensity in negative channels was the same across single-stain control samples. All analysis of flow cytometry data was performed using Cytobank Community (Kotecha et al., 2010). Sorting of APL cells for both DNA content and CD11b expression was performed with a BD FACSAria Fusion.

#### Immunoblotting

Protein was isolated from whole cell lysates using RIPA buffer (50 mM Tris-HCl, 150 mM NaCl, 1% Triton-X 100, 0.5% sodium dodecyl sulfate) supplemented with 1X Halt Protease Inhibitor Cocktail (Thermo Scientific 78430). The concentration of protein in lysates was quantified using a bicinchoninic acid assay (Thermo Scientific 23225). Lysates were diluted in LDS Sample Buffer (Invitrogen NP0007), reduced with 5 mM TCEP-HCl (Sigma-Aldrich 646547), and diluted to 0.4 mg/mL with deionized water to ensure equal loading. The samples were then boiled for 10 minutes before being electrophoretically separated on NuPAGE Bis-Tris gels (Thermo Scientific NP0335PK2). Proteins from the gel were then transferred to a nitrocellulose membrane (Thermo Scientific 88013). After transfer, the membrane was blocked for 1 hr with 5% non-fat milk in 1x Tris-buffered saline + 0.1% TWEEN 20 (Sigma-Aldrich P9416) (1x TBS-T), then stained with primary antibodies against Lamin A (Invitrogen MA1-06101) and GAPDH (CellSignaling 2118S) diluted 1:1000 in 5% non-fat milk in 1x TBS-T overnight at 4°C. The membrane was then washed three times for 10 minutes with 1x TBS-T, then stained with anti-mouse (LI-COR 926-68070) and anti-rabbit (LI-COR 926-32211) secondary antibodies diluted 1:10,000 in 5% non-fat milk in 1x TBS-T for 1 hr. The membrane was then washed three times with 1x TBS-T before being imaged on a LI-COR Odyssey.

#### RNA-Seq and differential expression analysis

RNA was extracted from ∼1 M cells using the QIAGEN RNeasy Mini Kit (QIAGEN 74104). A total of 36 samples were prepared: for each of three cell lines (AP-1060, HL-60, and NB4), the four conditions of untreated, ATRA, TSA, and ATRA & TSA were run in triplicate. Libraries were prepared using NEBNext Ultra II RNA Library Prep Kit for Illumina (New England Biolabs E7770S) and the NEBNext Poly(A) mRNA Magnetic Isolation Module (New England Biolabs E7490S) with 1 μg total RNA input. Paired-end 2 x 100 bp sequencing was performed on three Illumina HiSeq 4000 lanes with twelve samples per lane for an average sequencing depth of 31M reads per sample. Transcripts were quantified using Salmon (version 0.10.0) (Patro et al., 2017) in mapping-based mode with the human reference transcriptome (Ensembl GRCh38 version 86) (Cunningham et al., 2019). Read counts were then converted to counts per gene using Bioconductor (version 3.7) (Huber et al., 2015) with the *tximport* package (Soneson et al., 2015). Differential expression was determined using *edgeR* (version 3.22.0) and *limma* (version 3.32.0) (Law et al., 2014; McCarthy et al., 2012; Phipson et al., 2016; Ritchie et al., 2015; Robinson et al., 2010). Significance in differential expression was then filtered by a log-fold change greater than 1 using the TREAT method (*treat* function from *limma*) (Mccarthy and Smyth, 2009). Principal component analysis was performed with the *scikit-learn* library in Python (Pedregosa et al., 2011), and gene set overlaps were computed using the Molecular Signatures Database (MSigDB) and its *Investigate Gene Sets* tools (Liberzon et al., 2011; Subramanian et al., 2005). Gene set enrichment analysis was carried out using GSEA 4.0.3, using 497 gene sets from GO Cellular Component (c5.go.cc.v7.2.symbols.gmt) (Mootha et al., 2003; Subramanian et al., 2005) after applying a maximum and minimum size threshold of 500 and 15 genes, respectively. For enrichment mapping, enriched gene set nodes were filtered using a false discovery rate cutoff of 25%, and edges were filtered using a similarity cutoff of 50% (Merico et al., 2010; Shannon, 2003).

#### High-speed imaging

100 mbar of nominal inlet pressure from an Elveflow OB1 pressure controller drove cells through a mechanoNPS device. Images were acquired using a Fastec IL-5 high-speed camera at 1500 frames per second with a 167 μs shutter speed. Movie playback was set to 50 frames per second.

### Quantification and statistical analysis

Statistical details of each experiment can be found in the figure legends and in Table S1. Sample sizes, *n*, represent the total number of cells analyzed for a condition. The number of biological replicates and devices for each condition are provided in the figure legends.

Statistical tests for mechano-NPS data were applied to measurements of both *wCDI* and recovery time. Significant differences were principally calculated using two-sample Student’s t-tests with a significance criterion of *α* = 0.05. For experiments involving multiple comparisons, test statistics were instead computed using a Tukey’s range test. Where applicable, a Bonferroni correction to the significance criterion (by default, *α* = 0.05) was made for experiments involving multiple comparisons. For statistical tests that failed to reject the null hypothesis, we denoted this in our figures with NS (no significance). P-values lower than the significance criterion (0.05) were denoted with asterisks: ∗*p* < 0.05, ∗∗*p* < 0.01, ∗∗∗*p* < 0.001, ∗∗∗∗*p* < 0.0001.

To quantify the power of all mechano-NPS statistical tests, we performed a *post hoc* power analysis on all statistical tests made for both *wCDI* and recovery time (see Table S1). The statistical power for each test given the measured effect size and sample size was calculated and reported. For tests not exceeding a power value of 0.80, we reported the minimum effect size needed for a statistical power of 0.80, as well as the measured effect size.

## Data Availability

• RNA-seq data has been uploaded to the NCBI GEO with the identifier [NCBI GEO: GSE175507]. Mechano-NPS data has been uploaded to figshare with the identifier [figshare: https://doi.org/10.6084/m9.figshare.14668542]. Other data and images will be shared by the lead contact upon request. • All original code for analyzing raw mechano-NPS data are included in this GitHub repository. Files relevant to this manuscript can be found in the “mNPS_package” folder with prefix "mNPS_" and suffix "Li". Additional files not specific to mechano-NPS versions can be found in the "accessory" folder. • Any additional information required to reanalyze the data reported in this paper is available from the lead contact upon request.
