# Supplementary material for: Mechanical phenotyping reveals unique biomechanical responses in retinoic acid-resistant acute promyelocytic leukemia
Source: iScience. 2022 Jan 15;25(2):103772. doi: 10.1016/j.isci.2022.103772 (PMC8814755; doi:10.1016/j.isci.2022.103772)
Supplement: Document S1. Figures S1–S7 and Table S1 [file mmc1.pdf]

**iScience, Volume *25***

## Supplemental information

# Mechanical phenotyping reveals unique biomechanical responses in retinoic acid-resistant acute promyelocytic leukemia

**Brian Li, Annie Maslan, Sean E. Kitayama, Corinne Pierce, Aaron M. Streets, and Lydia L. Sohn**

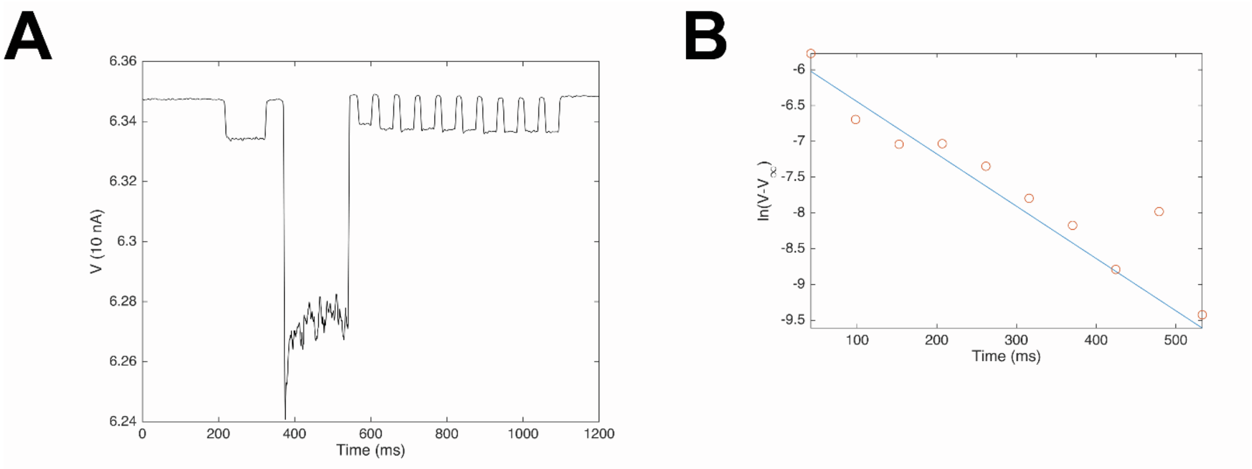


**Figure S1. MATLAB processing of mechano-NPS signals for quantifying cell recovery from deformation, related to Figure 1.**

**A.** Pulses are pre-processed as previously published [Kim *et al*. 2018], using a low-pass filter, base-line normalization, and derivative threshold to identify the start and end of each subpulse **B**. The logarithm of voltage values for each recovery segment subpulse (red circles) are fit to a linear function (blue line) using linear least squares regression (see STAR Methods).

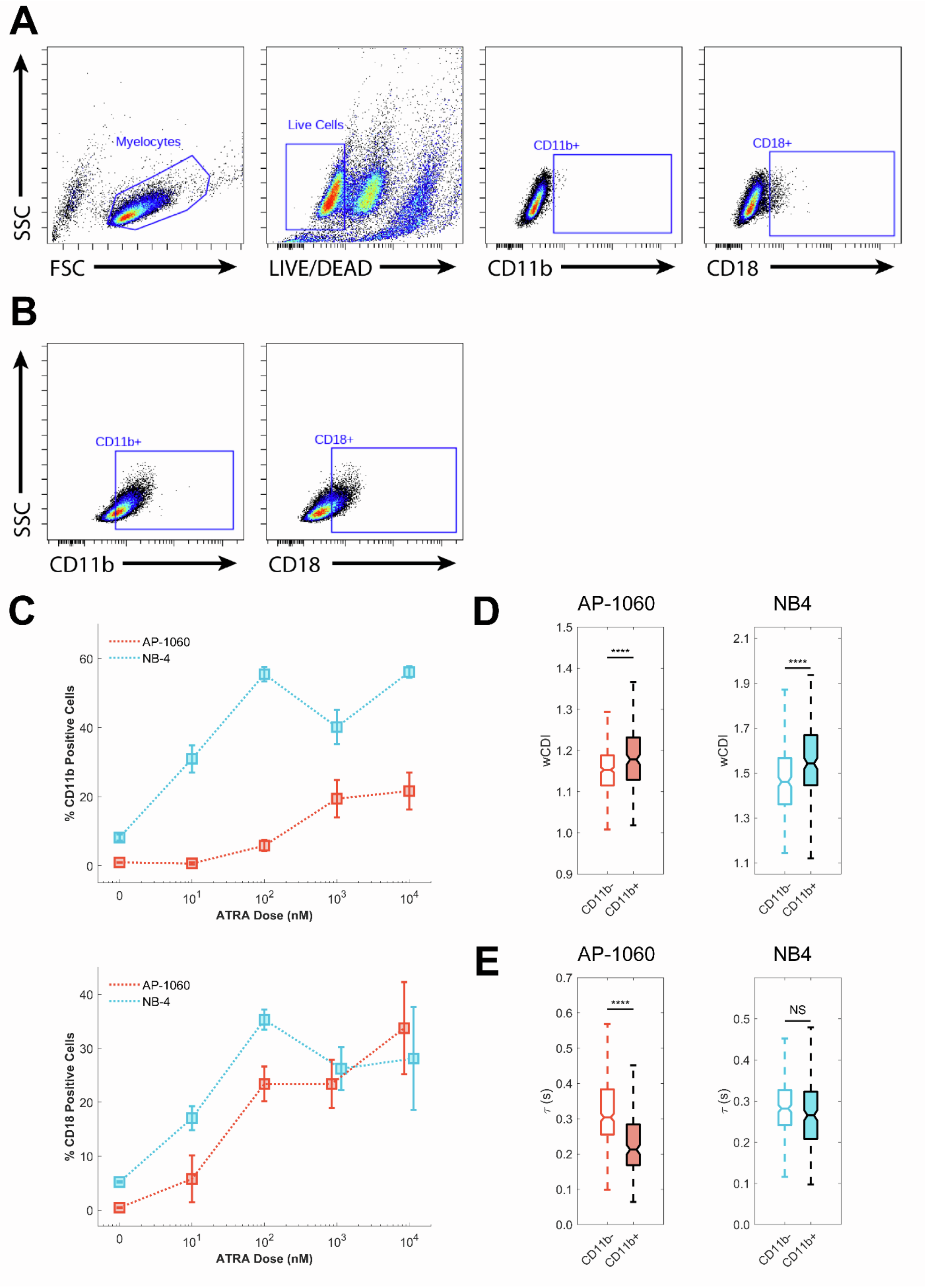
A
SSC
FSC
Myelocytes
Live Cells
LIVE/DEAD
CD11b+
CD11b
CD18+
CD18
B
SSC
CD11b+
CD11b
CD18+
CD18
C
AP-1060
NB-4
% CD11b Positive Cells
ATRA Dose (nM)
AP-1060
NB-4
% CD18 Positive Cells
ATRA Dose (nM)
D
AP-1060
NB4
wCDI
****
****
CD11b-
CD11b+
E
AP-1060
NB4
τ (s)
****
NS
CD11b-
CD11b+

**Figure S2. Flow cytometric analysis of APL cell line response to ATRA, related to Figure 2.**

**A, B.** Gating strategy for flow cytometry measurements of the CD11b and CD18 expression of AP-1060 and NB4 cells induced to differentiate with ATRA (representative plots from cells treated with 1 μM ATRA for 4 days). **A.** Cells are first identified as a cluster in side scatter (SSC) v. forward scatter (FSC). Live cells are then gated by the LIVE/DEAD-low cluster based on fluorescence from the LIVE/DEAD Violet dye. Fluorescence-minus-one (FMO) controls for each marker were used to determine the true negative fluorescence intensity distribution for the markers' respective fluorophores. **B.** Using gates established by FMO controls, the proportion of ATRA-differentiated cells expressing a certain marker can be measured. **C.** Proportions of cells positive for CD11b and CD18, markers associated with mature/differentiated innate immune cells, after treatment with varying doses of ATRA. A stronger differentiation response is seen in NB4 cells, indicating a higher susceptibility to induced differentiation via ATRA. Boxes and error bars represent the mean and standard deviation across 3 biological replicates. **D, E.** Box plots of *wCDI* (**D**) and recovery times (**E**) for AP-1060 (left) and NB4 (right) cells treated with 1 μM ATRA for 4 days and sorted for CD11b expression. Notches represent 95% confidence intervals for the true median of each distribution. AP-1060 CD11b- $n = 343$; AP-1060 CD11b+ $n = 191$; NB4 CD11b- $n = 234$; NB4 CD11b+ $n = 212$ measured on 3 different devices. ****$p < 0.0001$, determined by two-sample Student's *t*-tests.

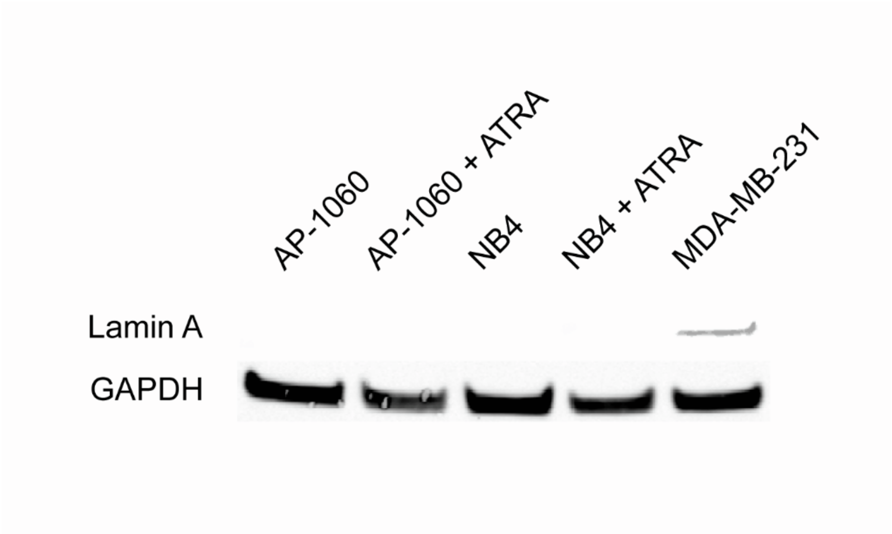


**Figure S3: Western blot for Lamin A in APL cells, related to Figure 2.**

Western blotting of protein isolated from whole-cell lysates of APL cells before and after ATRA treatment. MDA-MB-231 whole-cell lysate was used as a positive control.

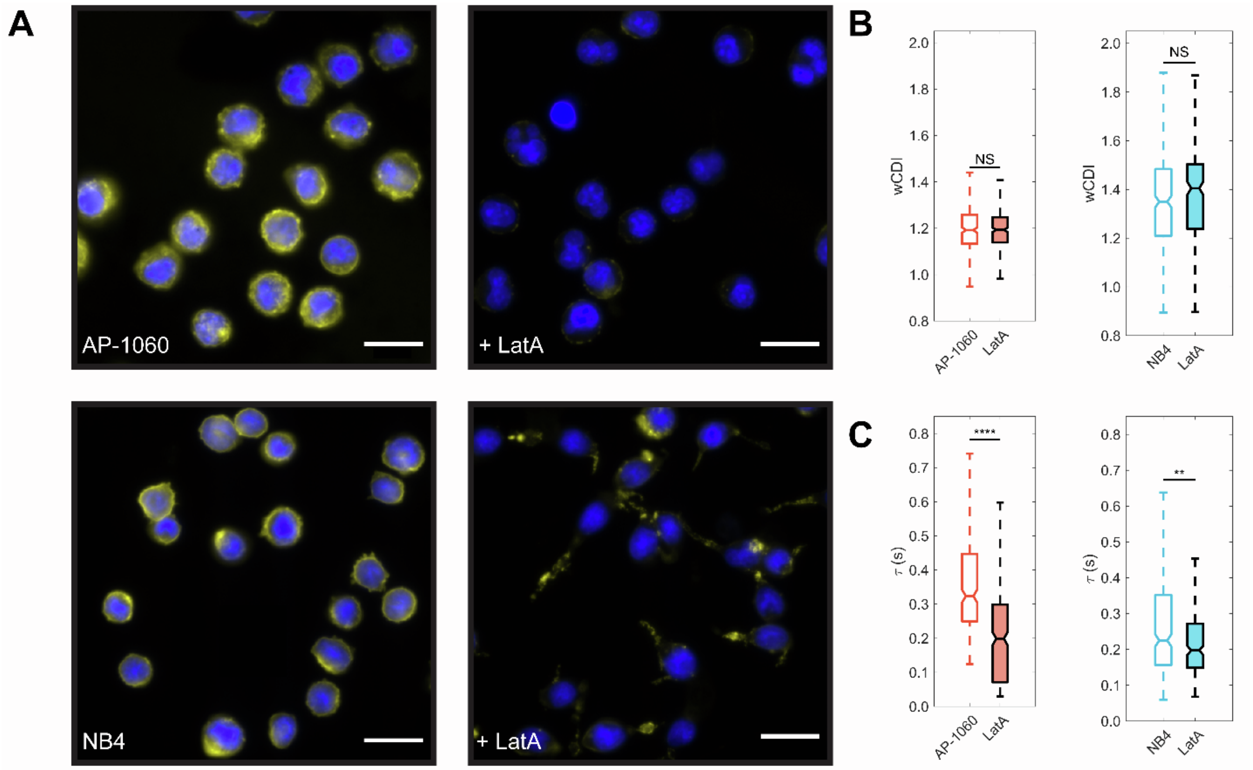


**Figure S4. Filamentous actin in APL cell cortices primarily influence cell recovery times, related to Figure 2.**

**A.** Fluorescence microscopy of AP-1060 and NB4 cells before and after treatment with Latrunculin A (LatA). Blue represents DNA stained with Hoechst 33342 dye, and yellow represents actin stained with rhodamine phalloidin. Scale bars represent 15 μm. **B, C.** Box plots of *wCDI* (**B**) and recovery times (**C**) for AP-1060 and NB4 cells before and after treatment with LatA. Notches represent 95% confidence intervals for the true median of each distribution. AP-1060 $n$ = 268; AP-1060 + LatA $n$ = 327; NB4 $n$ = 341; NB4 + LatA $n$ = 239 from 3 biological replicates each measured on a different device. **$p$ < 0.01 ****$p$ < 0.0001, NS no significance; determined by two-sample Student's $t$-tests.

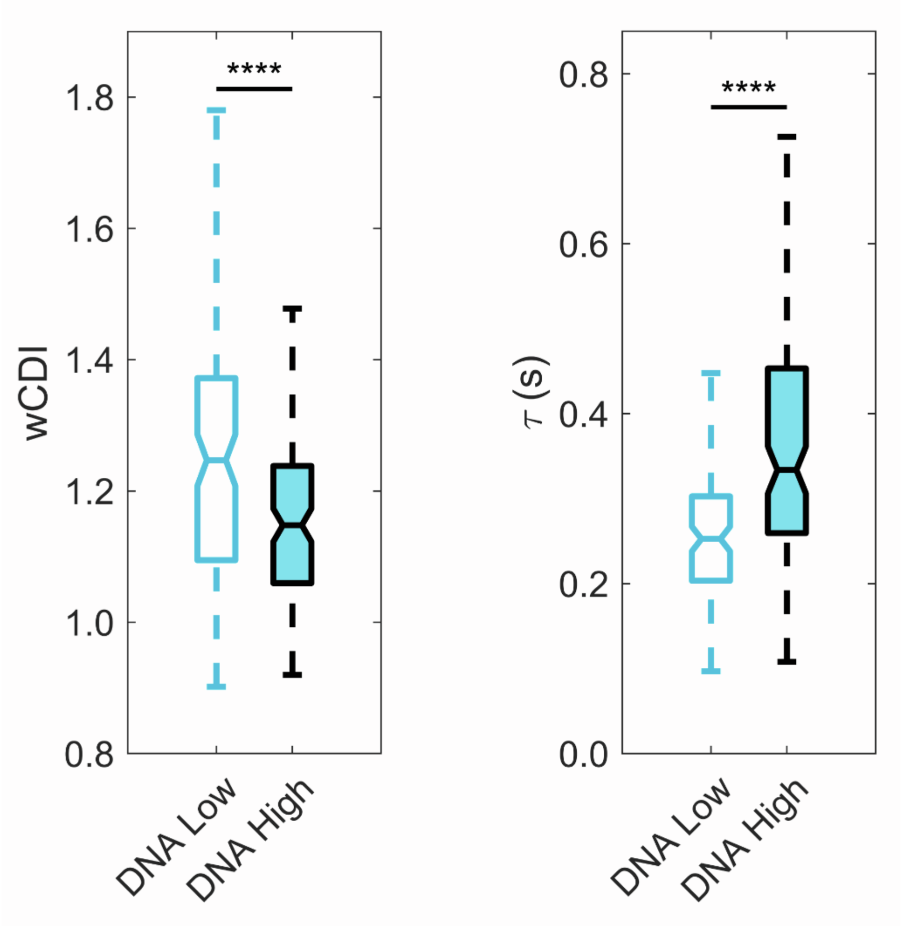


**Figure S5. NB4 cells sorted for DNA content demonstrate inverse relationship between DNA content and cell pliability, related to Figure 3.**

Box plots of *wCDI* (left) and recovery times (right) for NB4 cells stained for DNA (Hoechst 33342) and sorted by DNA content. NB4 populations were sorted into a “DNA Low” tube, indicating non-dividing cells, or a “DNA High” tube, indicating cells undergoing division at the time (M-phase). Cells from each tube were the analyzed with mechano-NPS. Notches represent 95% confidence intervals for the true median of each distribution. DNA low $n$ = 125; DNA high $n$ = 123 measured on 3 different devices. ****$p < 0.0001$, determined by two-sample Student’s $t$-tests.

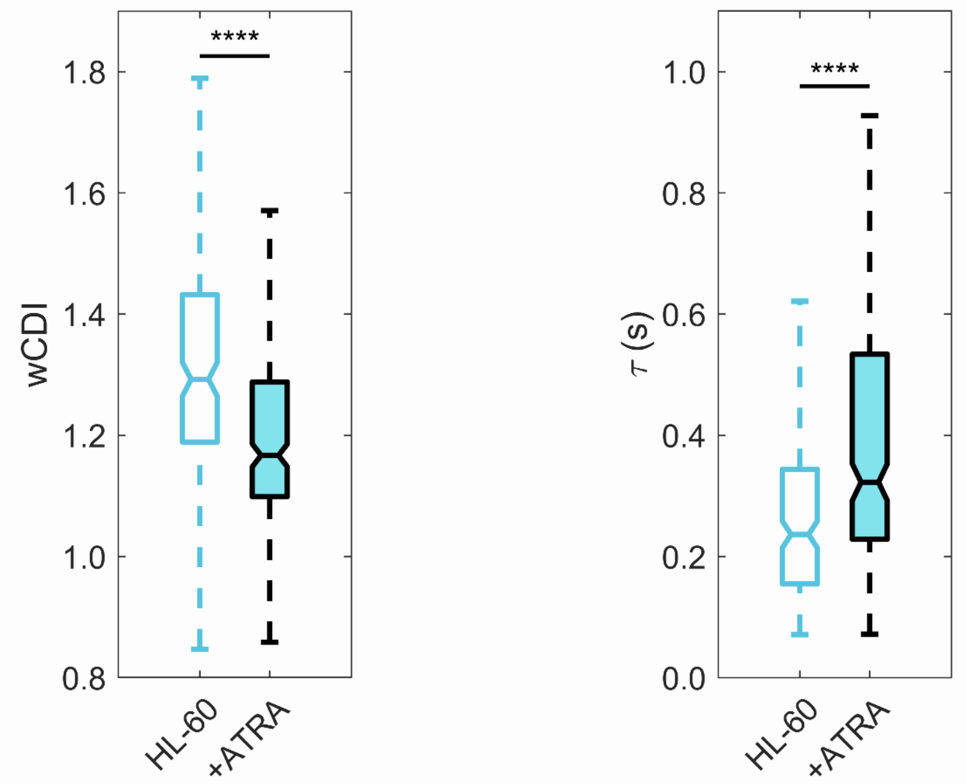


**Figure S6. HL-60, a non-APL cell line, treated with ATRA related to Figure 5.**

Box plots of *wCDI* (left) and recovery times (right) for HL60 cells before and after ATRA treatment. Notches represent 95% confidence intervals for the true median of each distribution. HL-60 $n$ = 184; HL-60 + ATRA $n$ = 252 from 3 biological replicates each measured on a different device. ****$p < 0.0001$, determined by two-sample Student's $t$-tests.

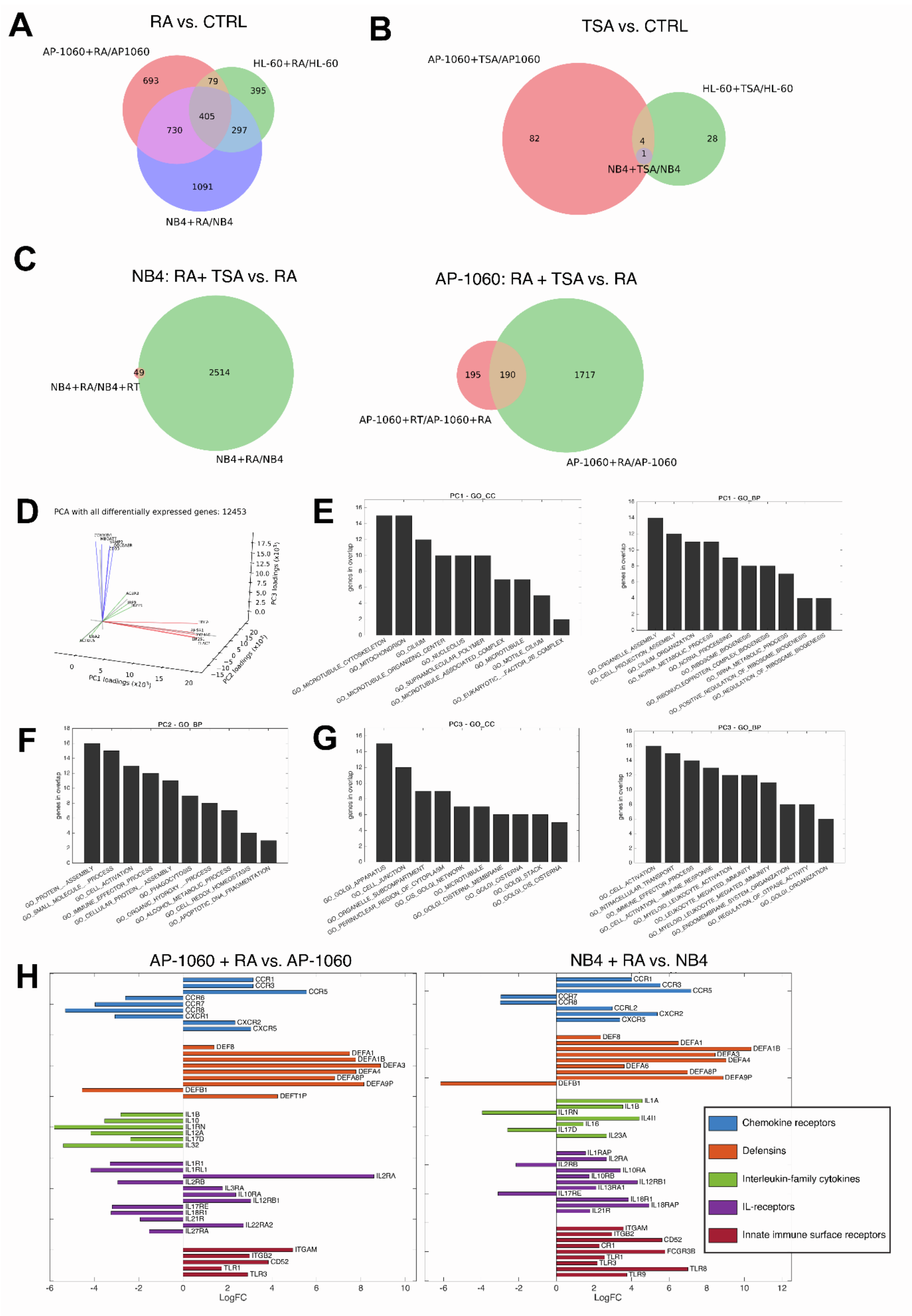
A
RA vs. CTRL
AP-1060+RA/AP1060
HL-60+RA/HL-60
NB4+RA/NB4
693
79
395
405
730
297
1091
B
TSA vs. CTRL
AP-1060+TSA/AP1060
HL-60+TSA/HL-60
NB4+TSA/NB4
82
4
1
28
C
NB4: RA+ TSA vs. RA
NB4+RA/NB4+RT
NB4+RA/NB4
49
2514
AP-1060: RA + TSA vs. RA
AP-1060+RT/AP-1060+RA
AP-1060+RA/AP-1060
195
190
1717
D
PCA with all differentially expressed genes: 12453
PC1 loadings (x10³)
PC2 loadings (x10³)
PC3 loadings (x10³)
E
PC1 - GO_CC
PC1 - GO_BP
genes in overlap
F
PC2 - GO_BP
G
PC3 - GO_CC
PC3 - GO_BP
H
AP-1060 + RA vs. AP-1060
NB4 + RA vs. NB4
LogFC
Chemokine receptors
Defensins
Interleukin-family cytokines
IL-receptors
Innate immune surface receptors

**Figure S7. Differential expression analysis across cell lines and treatment groups, related to Figure 5.**

**A-C.** Venn diagrams for differentially expressed genes identified via RNAseq. **A, B.** Untreated AP-1060, NB4, and HL-60 cells were compared to respective ATRA-treated samples (**A**) or TSA-treated samples (**B**). The sets of differentially expressed genes from each cell line-specific pairwise comparison were then compared. **C.** Comparisons to determine what effect TSA has on gene expression, in addition to or in interaction with ATRA. The set of differentially expressed genes for ATRA-treated vs. untreated samples was compared to that of ATRA- and TSA-treated vs. ATRA-treated samples for NB4 (left) and AP-1060 (right). **D.** Biplot for top 5 genes of each principal component according to PC loadings. **E, F, G.** Gene set overlap between the top 100 highest-loading genes of PC1 (**E**), PC2 (**F**), and PC3 (**G**) and MSigDB C5 (gene ontology) gene sets for GO cellular component (GO_CC) and GO biological process (GO_BP). For PC2, only one GO cellular component gene set (GO_NUCLEAR_OUTER_MEMBRANE_ENDOPLASMIC_RETICULUM_MEMBRANE_NETWORK, 13 overlapping genes) overlapped with input genes. **H.** Differentially expressed genes related to immune function and immunophenotyping identified in comparing ATRA-treated vs. untreated AP-1060 (left) and NB4 (right).

**Table S1. Power analysis for *wCDI* and recovery time constant by mechano-NPS experimental groups, related to Figures 2-4, S2-S6.**

| | | | | *wCDI* | | | Recovery time constant (ms) | | |
|---|---|---|---|---|---|---|---|---|---|
| Group 1 | Group 2 | $N_1$ | $N_2$ | Power | Min. effect size | Actual effect size | Power | Min. effect size | Actual effect size |
| **Figure 2** | | | | | | | | | |
| AP-1060 | NB4 | 246 | 124 | 1.00 | N/A | N/A | 1.00 | N/A | N/A |
| AP-1060 | + ATRA | 246 | 333 | 0.08 | 0.033 | 0.012 | 0.96 | N/A | N/A |
| AP-1060 | + ATRA + ATO | 246 | 424 | 1.00 | N/A | N/A | 1.00 | N/A | N/A |
| AP-1060 + ATRA | + ATRA + ATO | 333 | 424 | 1.00 | N/A | N/A | 1.00 | N/A | N/A |
| NB4 | + ATRA | 124 | 402 | 1.00 | N/A | N/A | 0.01 | 27.7 | 1.09 |
| NB4 | + ATRA + ATO | 124 | 419 | 0.03 | 0.049 | 0.014 | 1.00 | N/A | N/A |
| NB4 + ATRA | + ATRA + ATO | 402 | 419 | 1.00 | N/A | N/A | 1.00 | N/A | N/A |
| **Figure 3** | | | | | | | | | |
| AP-1060 | + Colcemid | 614 | 236 | 1.00 | N/A | N/A | 0.13 | 33.6 | 9.64 |
| NB4 | + Colcemid | 123 | 167 | 1.00 | N/A | N/A | 1.00 | N/A | N/A |
| AP-1060 | S-phase | 268 | 282 | 1.00 | N/A | N/A | 1.00 | N/A | N/A |
| AP-1060 | S-phase + Colcemid | 268 | 209 | 1.00 | N/A | N/A | 1.00 | N/A | N/A |
| AP-1060 S-phase | S-phase + Colcemid | 282 | 209 | 0.80 | 0.032 | 0.032 | 0.90 | N/A | N/A |
| NB4 | S-phase | 341 | 122 | 0.98 | N/A | N/A | 0.70 | 62.0 | 55.8 |
| NB4 | S-phase + Colcemid | 341 | 151 | 0.95 | N/A | N/A | 0.07 | 55.7 | 16.3 |
| NB4 S-phase | S-phase + Colcemid | 122 | 151 | 0.05 | 0.10 | 0.02 | 1.00 | N/A | N/A |

**Figure 4**

| | | | | | | | | | |
|---|---|---|---|---|---|---|---|---|---|
| AP-1060 Sync + LatA | + TSA | 155 | 84 | 1.00 | N/A | N/A | 0.27 | 81.4 | 54.1 |
| AP-1060 Sync + LatA | + ATRA | 155 | 199 | 1.00 | N/A | N/A | 0.29 | 52.6 | 29.9 |
| AP-1060 Sync + Lat A + TSA | + ATRA | 84 | 199 | 1.00 | N/A | N/A | 0.11 | 42.2 | 15.2 |
| NB4 Sync + LatA | + TSA | 75 | 114 | 0.68 | 0.094 | 0.083 | 0.44 | 55.9 | 38.9 |
| NB4 Sync + LatA | + ATRA | 75 | 95 | 0.79 | 0.103 | 0.101 | 0.47 | 61.3 | 43.8 |
| NB4 Sync + LatA + TSA | + ATRA | 114 | 95 | 0.09 | 0.058 | 0.019 | 1.00 | N/A | N/A |

**Figure S2D, E**

| | | | | | | | | | |
|---|---|---|---|---|---|---|---|---|---|
| AP-1060, CD11b- | AP-1060, CD11b+ | 343 | 191 | 0.99 | N/A | N/A | 1.00 | N/A | N/A |
| NB4, CD11b- | NB4, CD11b+ | 234 | 212 | 0.99 | N/A | N/A | 0.25 | 25.2 | 13.4 |

**Figure S4**

| | | | | | | | | | |
|---|---|---|---|---|---|---|---|---|---|
| AP-1060 | + LatA | 268 | 327 | 0.05 | 0.023 | 0.002 | 1.00 | N/A | N/A |
| NB4 | + LatA | 341 | 239 | 0.13 | 0.055 | 0.016 | 0.80 | 38.2 | 38.0 |

**Figure S5**

| | | | | | | | | | |
|---|---|---|---|---|---|---|---|---|---|
| NB4 DNA Low | NB4 DNA High | 125 | 123 | 0.92 | N/A | N/A | 1.00 | N/A | N/A |

**Figure S6**

| | | | | | | | | | |
|---|---|---|---|---|---|---|---|---|---|
| HL-60 | + ATRA | 184 | 252 | 1.00 | N/A | N/A | 1.00 | N/A | N/A |

*Post-hoc* power analysis was performed on all statistical tests for mechano-NPS measurements of *wCDI* and recovery time constant. Table rows correspond to specific comparisons made between experimental Group 1 and Group 2 with respective sample sizes $N_1$ and $N_2$. Bonferroni corrections to the default significance criterion $\alpha$ = 0.05 were made for experiments with multiple comparisons. For tests with statistical power equal to or less than 0.80, a minimum effect size for $\pi$ = 0.80 and actual effect size are reported.
